# Paths to language development in at risk children: a qualitative comparative analysis (QCA)

**DOI:** 10.1186/s12887-019-1449-z

**Published:** 2019-04-05

**Authors:** Kate Short, Patricia Eadie, Lynn Kemp

**Affiliations:** 1 School of Public Health and Community Medicine, University of NSW, Sydney, NSW Australia; 2 Graduate School of Education, University of Melbourne, Parkville, Victoria Australia; 3 School of Nursing and Midwifery, Western Sydney University, Sydney, NSW Australia; 4 Ingham Institute of Applied Medical Research, Liverpool, NSW Australia

**Keywords:** Children at risk, Language development, Risk and protective factors, QCA

## Abstract

**Background:**

Childhood language development is related to long term educational, employment, health and social outcomes. Previous research identifies a complex range of risk and protective factors which result in good and poor language outcomes for children, however children at risk are an underrepresented group in these studies. Our aim is to investigate the combinations of factors (paths) that result in good and poor language outcomes for a group of 5 year old children of mothers experiencing adversity.

**Methods:**

This mixed methods study utilised longitudinal data from a randomised control trial of sustained home visiting (MECSH) to determine the language outcomes in at risk children. Mothers were randomly assigned to a comparison group at entry to the study (prior to child’s birth). Their children who were retained at entry to school completed language assessments (*n* = 41) and were participants in this study. Influence of 13 key factors derived from the extant literature that impact language development were explored. Regression was used to determine the six key factors of influence and these were used in the Qualitative Comparative Analysis (QCA). QCA was employed to examine the necessary and sufficient conditions and paths affecting language development linked to good and poor language outcomes. A post hoc analysis of the risk and protective paths to good and poor language outcomes was also conducted.

**Results:**

Thirteen distinct pathways led to good language outcomes and four paths to poor language outcomes in five year old at risk children. A variety of condition combinations resulted in these outcomes, with maternal responsivity, toddler development and number of children in the home being key. High and low maternal education influenced both good and poor language development.

**Conclusions:**

The paths to good and poor language outcomes were different and complex. Most paths to a good language outcome involved protective factors, though not always. In addition, paths to poor language more often involved risk factors. The varied patterns of risk and protective factors point to the need for interventions across the first five years of life in both health and education for families which can respond to these risk and protective patterns.

**Trial registration:**

The original RCT was retrospectively registered in the ANCTR: ACTRN12608000473369.

**Electronic supplementary material:**

The online version of this article (10.1186/s12887-019-1449-z) contains supplementary material, which is available to authorized users.

## Background

Language use in children, the development of understanding and expression of words, grammar and discourse is one of the key and most complex developmental skills acquired through childhood, with far reaching affects through life. Good language development in children is important for effective long term academic, social and economic participation in society [1–3]. Poor language outcomes are evident from school entry [4, 5] and influenced by social determinants of health such as socioeconomic status (SES) and maternal education [6]. For this reason, language development is a focus of both public health and early childhood policy and practice [7].

### Language development and at risk children

Difficulties in language and communication place a burden on the child, parent and society both financially [8] and in quality of life [9]. One in five children have poor language development at 4 years [10] and there is a social gradient to the prevalence of language difficulties [11]. Children experiencing social disadvantage (as defined by census based measures of disadvantage e.g. income, suburb, home ownership, parental employment) are twice as likely as other children to have communication difficulties at entry to school [12] and language difficulties are part of both the cause and consequence of long term disadvantage. For some children, language difficulties lead to a sequelae of cascading negative effects such as poor literacy and social participation, increased academic failure, disengagement from school, instances of juvenile incarceration, a variety of mental health difficulties, generally poorer health, reduced employment and/ or increased relationship breakdown in adulthood [2, 3, 13–21]. Often these outcomes are clustered in children who come from low socioeconomic households and have parents who present with risks which result in these children being exposed to more difficult circumstances in their childhood [22]. This group we will refer to as *children who are at risk.* At risk here means an exposure to a combination of risk factors that have been shown to affect child development such as: low socioeconomic status, limited resources parental capacity and /or physical needs such as housing, experiencing mental health and drug and alcohol difficulties in the home, child maltreatment and domestic violence amongst other stressors and threats [23].

### Risk and protective factors for language development

Utilising a bioecological model of development [24], research has established some of the key child, maternal and environmental influences on language development in the early years. These include (but not exclusively) maternal factors such as education [25], mental health [26–28] and responsivity [29], a family history of communication difficulties [30]; child factors such as birth weight [31], toddler development [32] and gender [33] and environmental factors such as being read to from an early age [34, 35], numbers of children in the home [36–38], attending sufficient good quality childcare [39] and SES [40–42]. Debate continues as to whether these factors are mediators or causal in language outcomes [43].

Though we know these risk and protective factors impact language development, longitudinal cohort studies have consistently shown individual factors on their own represent only small amounts of the variability in language skills [30, 44–46]. It has been found there is a compounding effect of multiple risk factors on vocabulary development [47]. However, how these risk and protective factors combine and impact on each other in language development has been less studied. In a recent example, Baydar and colleagues (2014) investigated the impact of a combination of multiple maternal and environmental family factors on vocabulary development in Turkish children. The responsivity of low SES mothers supported children’s vocabulary development only when the mothers were not depressed. Investigations of risk “clusters” for language development have also emerged. In a longitudinal study of Australian children, those with a risk profile related to speaking a language other than English made fast gains in language through the school years if they had few other risks. However, if they had a number of risks, both the English and non-English speaking children performed poorly. Those with many risks for poor language at the end of the study performed poorest. [47]

However, not all children with risks end up with language difficulties. What protects against poor language has received some limited attention and reveals some key factors. Turkish children’s vocabulary development was protected in families of depressed mothers who were economically distressed if they were surrounded by a supportive family and community [27]. Other studies of population and impaired cohorts have found being regularly read to, attending early childhood education, participating in play and the child’s prosocial skills at 4 years were all protective [7, 48].

### Interventions targeting risk and protective factors

As there are so many possible sources of influence on language development, both proximal and distal to the child, determining the most influential conditions will help services target and create public health preventative interventions [4, 35]. Some conditions such as book reading can be changed through interventions or are manipulable. The environmental impacts that may be manipulable in interventions play an important role in the early years of language acquisition and provide an opportunity to prevent language difficulties or change the trajectory of development for some children [12, 35, 49]. Brofenbrenner and Morris [50] outline there is an interdependence between conditions. Influencing one of these conditions can have an effect on others, these factors being both producers and products of development. There is research, community and governmental interest in targeting low SES groups, where children with more manipulable risks appear to be concentrated and there are some promising interventions documented [12, 51] . However, it is unclear, how one or many of these risk and protective conditions should be targeted in interventions to create best language outcomes. Further research is required to explore the combinations of risk and protective factors in at risk children to help develop more tailored interventions for this population.

### Statement of the problem

Large prospective cohort studies which unpack key conditions that predict future language abilities are numerous (see Law, Dennis (43) for a review). The factors relate in complex, non linear ways with each other and a number of them interact and reinforce each other [25]. Considering just one of these and trying to “partial out” its influence is conceptually difficult. Qualitative Comparative Analysis (QCA) is a method designed to help unpack these complex relationships, however it is a method which to our knowledge, is untested in the realm of child development. QCA is a mixed method standing between qualitative and quantitative methodologies and has been employed to help answer complex health policy questions [52]. Blackman et al. (2013) explains QCA as “… particularly apt for producing evidence about how to tackle complex policy problems that have the character of ‘wicked issues’ … These are issues that pose significant challenges for intervention because of interdependencies between causes.” (pg. 127). Language development in at risk children is one such “wicked issue”. In this study we use QCA to explore the specific *combinations* of risk and protective conditions important for good and poor language outcomes in a low SES, culturally and linguistically diverse group of 5 year old Australian children at risk of compromised child development. We hypothesize that different factors combine in complex ways to create good and poor language outcomes.

## Methods

This mixed method, prospective cohort study was nested within the Miller (subsequently Maternal) Early Childhood Sustained Home visiting (MECSH) randomised control trial [53]. MECSH explored the effectiveness of sustained, nurse home visiting provided to women experiencing adversity from pregnancy until their child was two years old We conducted secondary, quantitative and qualitative analysis of a range of data collected over the 5 ½ years of the study, exploring whether there were multiple paths to good and poor language outcomes. This study comprises of data from the comparison arm of the RCT as they represent a population non-intervention group who received usual care. Usual care at the time meant a mother received a home visit by a child health nurse within 2 weeks of giving birth with the offer if subsequent visits to a well child clinic if the mother chose to attend. All study participants gave written informed consent prior to entering the study.

### Recruitment

**Fig. 1:**
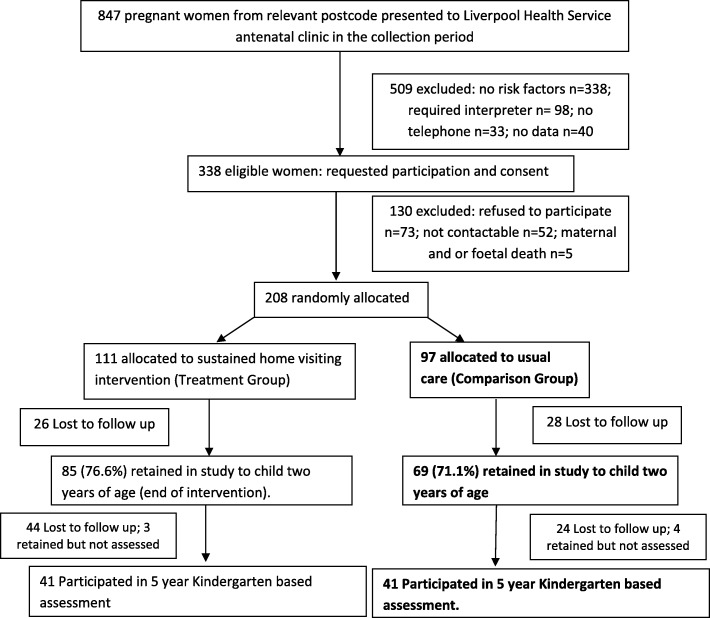
Recruitment and retention for the MECSH RCT and this study

208 low SES, mothers experiencing adversity were recruited from the Liverpool Hospital, New South Wales. These women were assessed on routine psychosocial assessment at their first presentation to the hospital antenatal clinic prior to the child’s birth. They were eligible for the study if they lived in a particular socio economically disadvantaged area and presented with one or more risk factors for poor maternal or child outcomes. These risks included: mental health problem or disorder (past or current); teenage parent; late antenatal care (after 20 weeks); current substance misuse; history of or current domestic violence; history of abuse as a child; lack emotional or practical support; major stressors in the last 12 months; current probable distress (as indicated by an Edinburgh Postnatal Depression Scale [54] score of 10 or greater). On consent, participants were randomised, 111 to treatment and 97 to comparison, trial number: ACTRN12608000473369 [53]. At 5 years (entry to school), 86 children (41%) were retained, 82 of these were assessed on the Wechsler Preschool and Primary Scale of Intelligence III (WPPSI-III) Australian Edition [55], and 41 of these children from the comparison group were eligible for the current study (see consort diagram Fig. 1).

Eligibility for this study included: the child completing a cognitive assessment (WPPSI-III) at end of the first term of formal school entry around 5 years of age (mean = 65 months, SD 4.3) by those in the original comparison group. QCA is a methodology suited to medium n sample sizes, with n between 10 and 50 being ideal [56], thus this sample is appropriate for this methodology. For this study, the retained comparison group participants (*n* = 41) were compared to the original comparison group (*n* = 97) (see Table 1). There has been an attrition of 59%, and there was one significant difference between the groups: significantly more single mothers were retained than there were in the original cohort (*p* = 0.022).

**Table 1:** Maternal and child characteristics original vs retained groups

Characteristic at child’s birth	Original comparison Group *n* = 97 (%)	Retained comparison Group *n* = 41 (%)	*P* values
Mean Maternal age (SD)	27.7 (5.91)	27.7 (5.29)	0.95
Parity n (%)			
first child	34 (35.1)	13 (31.7)	0.705
second or later child	63 (64.9)	28 (68.3)	
Maternal Country of Birth n (%)			
Australia	50 (51.5)	26 (63)	0.200
Not Australia	47 (48.5)	15 (37)	
Marital Status n (%)			
married / living with partner	79 (84.9)	27 (67.5)	0.022*
single / separated/ divorced	14 (15.4)	13 (32.5)	
Level of Education n (%)			
High School / vocational	74 (80.4)	33 (82.5)	0.781
Degree or higher	18 (19.6)	7 (17.5)	
Household income source n (%)			
Full or part time wages	66 (72.5)	32 (80.0)	0.364
Benefit or pension	25 (27.5)	8 (20.0)	
Housing Tenure n (%)			
Own or purchasing	43 (50.6)	22 (57.9)	0.452
Renting or other	42 (49.4)	16 (42.1)	
Presenting psychosocial risks			
One risk	49 (50)	22 (54)	0.736
Two or more risks	48 (50)	19 (46)	
Depression	43 (44)	13 (32)	0.168
Mental health problems	30 (31)	17 (41)	0.233
Late antenatal care	35 (36)	9 (22)	0.356
Stressors	33 (34)	14 (34)	0.189
History of abuse	13 (13)	9 (22)	0.2099
Teen mother	7 (7)	3 (7)	#
Experiencing domestic violence	4 (4)	1 (2)	#
Drug and alcohol abuse	1 (1)	1 (2)	#

*significant at 0.05; # Note no p values calculated due to the limited numbers in each category

### QCA method, theoretical and methodological framework

The QCA process is iterative (thus qualitative in nature) but structured in its method and relies on the principles of Boolean logic and set theory [57]. It compares the empirical evidence of individual cases with all theoretically possible combinations of risk and protective factors (paths) that lead to an outcome. Through a process of logical minimisation, only those conditions that clearly differentiate good vs poor outcomes are retained in the final explanatory models.

In QCA, 3 key phenomena further our knowledge of what causes an outcome: conjunctual causation, equifinality and causal asymmetry. [58]. *Conjunctual causation* refers to the specific *combinations* of causes lead to a specified outcome. Prior evidence and theory help determine the combinations of risk and protective conditions to place in cumulative risk models. QCA then uses empirical cases to investigate which conditions and in risk or protective mode combine to result in an outcome. In QCA this result is called a *path* or *causal recipe* [57]. There may be a variety of these causal recipes which result in the same outcome. This leads to the second important and novel contribution QCA provides - the notion of *equifinality* [57]. Equifinality refers to an expectation that there can be multiple paths that lead to an outcome. QCA may thus move us closer to understanding the multiple combinations of risk and protective factors which result in and good and poor language outcomes. In turn, understanding these complex paths may help us develop more effective interventions.

Finally, investigating *causal asymmetry* is an important methodological task of QCA. Causal asymmetry assumes good and poor language outcomes are not the result of paths that are the direct opposite of each other.

The two mathematical concepts of necessity and sufficiency are investigated in QCA and are essential to understanding results. Each variable, called a condition in QCA, may be necessary or sufficient (or neither) for an outcome. For a condition to be necessary it must be present for the outcome to come about. Necessity gives clear instructions for intervention. However, few conditions are usually necessary. More common is that conditions are *sufficient.* That is, when a condition is combined with one or more conditions it is causal for the outcome [56]. Examination of the sufficient condition combinations will help develop more targeted language interventions for at risk children and families.

### QCA processes

QCA has two main steps: 1. Qualitative analysis of cases when the data set is cleaned and calibrated (the process of deciding the rules by which presence or absence of the condition is determined for the outcome and each condition) and 2. Quantitative stage when there is comparison across cases and all possible paths to an outcome are configured through Boolean minimisation. There are two possible methods of QCA crisp set (csQCA) and fuzzy set (fsQCA). In csQCA, all conditions are binomially split, into the “presence” or “absence” of the condition where as in fsQCA the more continuous data is retained. Crisp set was chosen for this first study of QCA with child language, as it allows exploration of the most simple path combinations [57]. The dichotimisation is part of the data analysis and is outlined for each condition in Table 2 and detailed in Additional file 1.

**Table 2:** Calibrated Outcome and Conditions, their codes and n for each group

Outcome	Measure/s	Set*	code	N	mean
Language Status at 5 years					
Good Language	WPPSI VIQ: Standard Score (SS) 85+	1	GL	33	107.1 (13.0)
	Teacher perception scale: 5+				6.57 (1.1)
Poor Language	WIPPSI VIQ:SS 84 & below	0	PL	8	88 (10.7)
	Teacher perception scale: 4 & below				5 (1.5)
Condition	Measure/s	Set*	code	N	mean
Child Gender					
Female		1	F	22	
Male		0	M	19	
Toddler Development	Bayley’s Mental Development Inventory (MDI) [78]				
Good	SS = 85+	1	D	32	103.8 (11.4)
Poor	1SD below mean (SS = 84 and less)	0	d	8	77.4 (6.2)
Behaviour at 3 years	Bayley’s Behaviour Scale: Percentile Rank (PR) [78]				
Good	within 1SD & above (PR 26 & above)	1	B	30	78.0 (19.0)
Poor	>1SD below mean (PR 25 & below)	0	b	11	12.2 (6.5)
Education	Age mother left school				
High	17 years+	1	ME	21	17.5 (0.51)
Low	16 years and below	0	me	18	15.6 (0.54)
Distress antenatal	Edinburgh Postnatal Depression Scale (EPDS)				
Not distressed	9 or under EDPS^ at recruitment	1	AD	28	4.3 (2.7)
Distressed	10 or over EPDS at recruitment	0	ad	13	11.8 (1.5)
Chronic Distress Overtime	EPDS & Center for Epidemiologic Studies Depression Scale (CES-D)^^^ over 5 time points				
Not chronically distressed	distress present > 50% of the time asked	1	CD	35	10.0% (14.7)
Chronically Distressed	< 50% of the times distress was measured the mother presented with a concerning score on either the EPDS or the CES-D^	0	cd	6	93.3% (16.3)
Maternal Responsivity	Home and clinic assessment of responsivity: HOME Responsivity Subscale [79] + play sample mother child interaction coding [80]				
Good	Scores above the sample means for HOME (9) &/or play sample (10)	1	RS	21	22.95 (2.2)
Poor	Scores below the sample means for HOME (8) and /or play sample (9)	0	rs	20	16.1 (2.6)
Number of children in the home	Parent report number of children in the home at study child’s birth				
Less	2 or less	1	CH	27	1.5 (0.5)
More	3+	0	ch	14	3.7 (1.2)
LOTE^$^	Languages spoken in the home as reported at birth, 18 and 33 months				
English	English only spoken at home	1	English	22	
LOTE	12 different Language/s Other Than English spoken at home. Most common: Arabic, Samoan and Spanish.	0	LOTE	19	
Family Origin	Recent migrant or not collected antenatally at recruitment				
	First generation migrant	1	FO	14	
	Not first generation migrant (First Australian Aboriginal, second generation or more migrant)	0	fo	27	
SES	Parent report of housing status and income source on 2 different occasions				
High	49% or less low housing and income	1	SES	21	
Low	50% of the time or more low housing and income	0	ses	20	
Read to 3 X / wk	Parent report number of days read to child on 3 different occasions (child 12 and 24 months of age and prior to school entry).				
Good Reading Amount	Presence of good consistent reading: Read to 3 times a week each time	1	RD	21	
Poor Reading Amount	Absence of good consistent reading: not read to 3 times on at least one occasion	0	rd	19	
Early Childhood Education (ECE)	Parents reported ECE attendance at 9 possible times from 12 months to just prior to starting school.				
Optimal ECE Amount	24+ months of centre based care	1	CC	19	26.2 (4.7) months
Non optimal ECE Amount	Absence of any centre based care &23 months or less of centre based care	0	cc	22	11.98 (7.5) months

*1 = protective 0 = risk; ^ EPDS: cut point of 10; CES-D: cut point of 16 as outlined in the MESCH coding guideline [81]; ^$^ LOTE: Language Other Than English

### Creating, cleaning and calibrating the dataset

#### Outcome: Language status at school entry (see Table 2)

Language outcome was based on the combination of both functional and standardised test performance in English, administered in the first year of formal schooling. The standardised test performance was determined by utilising the language quotient on the WPPSI-III [55] verbal subscale (VIQ) which contained 3 subtests of language skills: Vocabulary, Information and Word Reasoning subtests administered at the children’s schools by a registered psychologist. A standard score of 85 or higher was set as good language outcome, 84 and below as poor language outcome. Functional performance was determined by teacher perception of children’s language skills: teachers rated the child’s expressive (spoken) and receptive (understanding) language skills separately in comparison to their peers on a 4 point Likert scale: 1. Much less competent 2. Less competent than others 3. As competent as others 4. More competent than others. The points from these teacher ratings were totalled and each child received a score (possible range 2–8) for their language skill at school and a score of 5 or higher was considered good language outcome, 4 and below for poor language outcome. For the QCA, a child was considered to have the outcome of Poor Language (PL) at 5 years if they fulfilled criteria for poor language in either measure. There were 8 children with PL (19.5% of the sample). All others were considered Good Language outcome (GL: *n* = 33) users. Further details of the creation and calibration of this outcome are available in the supplementary file (Additional file 1).

#### Conditions (see Table 2)

The conditions for the QCA were selected if they were: (1) identified in the literature as salient to child language outcomes from birth - entry to school; (2) represented child, maternal and or environmental risks and protections; (3) able to be divided for analysis in a logical method with at least 25% of the sample meeting criteria for binary categories [56] and (4) available in the MECSH dataset. This resulted in 13 conditions (see Table 2) which were: *Child*: gender (G); toddler development (D) and behaviour (B). *Maternal*: education (ME); antenatal distress (AD); chronic distress overtime (CD); responsivity in infancy and toddlerhood (RS). *Environmental*: Socioeconomic Status (SES); number of children in the home (CH); child read to more than three times a week over time (RD); two years of more of early childhood education prior to starting school (ECE); Language spoken: Language other than English (LOTE) or English.

Detailed discussion of how the conditions were operationalised and calibrated is outlined in Additional file 1. A range of parent reported survey data, child and parent assessments and coding of videos were used to create the categories that defined the conditions. All conditions were then binomially cut, with the cut point informed by literature, standardised score/test manual recommendations or natural divisions in the data. Some conditions were simple compilations of data with clear cut points, for example the standardised score from the Bayleys MDI was used for the child toddler development (D) condition and cut at 1 SD below the mean (Poor development). Some conditions were more complex to establish and a variety of longitudinal data were used to create them. For example, to form the condition maternal responsivity (RS), data from two different sources at two different time periods were used. Quality of maternal responsivity was operationalized combining a home based analysis using the responsivity subscale score from the HOME Inventory [59] and NICHD rating of maternal-child interaction in play sample videos conducted in the clinic [60]. The mean of each of the play sample scores and the HOME responsivity rating were set as cut points and any child below the mean on either one of the measures received a score of zero (condition absent) for responsivity.

Each condition was coded for every participant, referred to as a case in QCA. All conditions were constructed in the positive, thus presence of a condition, meant a case was assigned a score of one for that condition and this indicated a notionally protective variable for good language development. Assigning zero meant absence of the condition or risk of lower language development. In all conditions, missing data fields were left unassigned.

The next process was to reduce the number of conditions to that which could be adequately supported by the number of cases, providing good model coverage [56]. Quantitative methods were utilised to reduce the number of conditions from 13 to the maximum of seven considered to be adequate with the number of empirical observations to maintain good diversity [57]. Initially correlation between the conditions was conducted, controlling for language outcome on the WPPSI standard score at 5 years. Two factors were highly correlated: behaviour and development. Development was chosen to remain in the set of predictors due to the stronger correlation with language outcome (development: r = 0.490, *p* = 0.001; behaviour: r = 0.312, *p* = 0.050). Regression was then used to determine those conditions with greatest predictive value for the language outcome at 5 years as measured on the VIQ of the WPPSI–III [55]. Each condition was individually regressed against the outcome and cut point of *p* = 0.1 or less was used for inclusion in further analysis.

Regression revealed seven significant factors predicting language outcome at 5 years for inclusion in the QCA (see Table 3): toddler development; maternal education; maternal antenatal distress; maternal responsivity in infancy and toddlerhood; number of children in the home; amount of early childhood education prior to starting school and Language Other Than English (LOTE) being spoken at home. Once the significant predictors were chosen a further three cases were excluded due to missing data. Thus the final group for analysis in QCA was 38 cases.

**Table 3:** Simple regressions for each predictor with the language outcome at 5 years

Individual Regressions		Variance R^2^	B (intercept)	Include in QCA
Child				
gender	t = 0.220 (*p* = 0.827)	1.0%	0.131	×
toddler development	t = 3.461 (p = 0.001)**	24.0%	0.468	✓
Maternal				
maternal education	t = −1.764 (*p* = 0.086)	8.0%	−3.335	✓
antenatal distress	t = 2.035 (*p* = 0.049)*	10.1%	−1.106	✓
chronic distress overtime	t = 1.459 (*p* = 0.153)	5.2%	−0.100	×
maternal responsivity	t = 3.601 (*p* = 0.001)**	25.0%	1.738	✓
Environmental				
LOTE^#^	t = 2.004 (*p* = 0.052)	9.3%	8.868	✓
SES	t = −0.47 (*p* = 0.963)	0.0%	−3.63	×
number of children in home	t = 1.870 (*p* = 0.069)	8.2%	−3.223	✓
early childhood education	t = 3.013 (*p* = 0.005)**	18.9%	12.61	✓
read to 3 times a week	t = −0.359 (*p* = 0.722)	3.0%	−2.408	×

** significant at 0.001; * significant at 0.05; ^**#**^*LOTE = Language Other Than English spoken at home*

### Path configuration and analysis

**Fig. 2:**
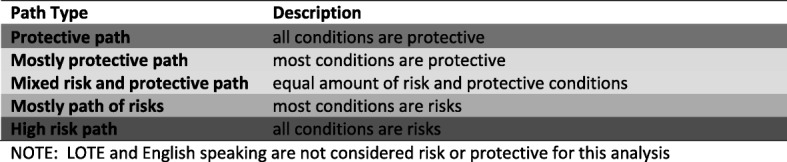
Classification of paths by combinations protection and risks conditions

Following choice of variables, the conditions were placed in the fsQCA 2.5 software program and two QCA analyses were conducted: one exploring paths to Good Language (GL) outcome and one the paths to the Poor Language (PL) outcome. A truth table was established, which contains all of the condition combinations logically possible. A minimum consistency of 0.75 and coverage of 0.5 were used as boundaries to determine sufficient paths [56]. Following this, the Quine-McCluskey algorithm which uses Boolean algebra to compute the commonalities between the paths that lead to GL and PL was used. 7This logically reduces the configurations to produce a solution [61, 62]. There are two parameters used to reduce rows: 1. Coverage: the empirical relevance of a solution; and 2. Consistency: the extent to which cases sharing similar conditions display the same outcome. Initially necessary condition analysis was conducted to determine those conditions which were essential to the outcome. Parameters of fit were set to determine necessary conditions these were: consistency 0.9 and coverage 0.5 [56]. The paths were then exposed to sufficiency analysis. This is a more complex analysis to determine if any conditions or combinations of conditions (conjunctual causation) were essential for either the Good or Poor Language outcome. There are three possible solution models to report in QCA. For this study the intermediate solution, a combination of both theory and empirical data will be presented below. This was chosen to allow both the empirical data and theory influence over the final solution. The other two possible solutions (the complex and parsimonious) can be found in the Additional file 1. Subsequently, further classification of the paths was conducted according to risk and protective components as outlined in Fig. 2.

## Results

The number of conditions predict the number of possible paths, thus in this study seven conditions predicted 128 different possible paths to Good (GL) and Poor Language (PL) outcomes, of which 32 were created by the empirical data (coverage 25%). Of these, 27 were paths to GL and five paths to PL (see Table 4). Most paths had one case however there were six paths with two or more cases. Two of these paths (which represented 4 of 38 cases) had cases with different outcomes (conflicts) (Paths 6 and 7 in Table 4). Note, in reporting of the paths an asterisk (*) represents a logical “AND”; upper case codes e.g. “D” for toddler development, indicate the protective form of toddler development and lower case “d” indicates the risk form of the condition. One path D*me*RS*ch*ece*LOTE, had an outcome of both PL (case 36) and GL (case 74) and the other path D*ME*rs*CH*ECE*LOTE also had an outcome of PL (case 73) and GL (case 44). These resulted in truth table conditions of 0.5 consistency which contravened the consistency boundaries and these four cases were removed from further analysis. To check for their influence, analysis were conducted with and without the cases. Excluding these cases resulted in a change of one path, with the addition of one condition (with the above cases Path 9 of GL was: D*AD*RS and without the cases was D*AD*RS*English). There were no changes to the PL solution with removal of the cases. Thus, there was minimal impact of removing the conflict cases, although these cases were explored further to develop understanding of the conflicting outcomes. All further analysis was conducted with these cases removed. As not all paths could be represented by the 38 cases, the empirically unrepresented paths (logical remainders) required consideration in the analysis. Logical remainders for five of the six conditions were set to present (for GL) or absent (for PL) in as indicated by the literature except LOTE / English speaking. This condition was set to neither present nor absent in both the GL and PL QCA. This was done as investigations of the data indicated LOTE speakers were equally present in both PL and GL outcomes. The truth table emerged as outlined in in the additional file (now without Paths 6 and 7). This led to the intermediate solution presented below.

**Table 4:** Paths to good and poor language outcome, coverage consistency, cases and risk type

Path	Good Language (GL) Path Formula	Coverage		Consistency	Cases	GL Path Types
		Raw	Unique			
1	d*me*English	0.07	0.035	1	13, 35	High Risk
2	D*M E*CH	0.4	0.1	1	20, 21, 26, 30, 51, 54, 55, 57, 60, 68, 69	Protective
3	AD*RS*CH	0.4	0	1	9, 13, 21, 46, 55, 60, 68, 69, 71, 79	Protective
4	me*AD*CH*LOTE	0.1	0.04	1	46, 47, 79	Mostly Protective
5	me*CH*LOTE*ECE	0.03	0.04	1	1	Mostly Protective
6	D*ME*AD*LOTE	0.1	0.04	1	54, 61, 69	Protective
7	D*ME*RS*LOTE	0.1	0.04	1	24, 30, 69	Protective
8	D*ME*LOTE* ECE	0.1	0.04	1	30, 41,69	Protective
9	D*AD*RS*English	0.3	0.1	1	9, 18, 21, 28, 55, 60, 66, 68, 71	Protective
10	me*AD*CH*ECE	0.1	0	1	3, 6, 9	Mostly Protective
11	me*RS*CH*ECE	0.1	0	1	9, 25, 72	Mostly Protective
12	D*AD*CH* ECE	0.2	0	1	3, 6, 9, 20, 55, 60, 69	Protective
13	D*RS*CH* ECE	0.2	0	1	9, 25, 30, 55, 60, 69, 72	Protective
solution coverage: 1; solution consistency:1.						
Path	Poor Language (PL) Path Formula	Coverage		Consistency	Cases	PL Path Types
		Raw	Unique			
1	d*ME*rs*LOTE*ece	0.4	0.2	1	45,64	Mostly path of risk
2	D*me *rs*ch*ENGLISH	0.2	0.2	1	31	Mostly path of risk
3	D* me *ad*rs*ch* ece	0.2	0.2	1	45, 52	Mostly path of risk
4	d*ME*ad *rs*ch*LOTE	0.4	0.2	1	38	Mostly path of risk
solution coverage: 1; solution consistency:1.						

D/d = good/poor toddler development; ME/me = high/low maternal: education; AD/ad = no/present antenatal distress; RS/rs = good/poor responsivity in infancy and toddlerhood; CH/ch = 1-2children / 3+ children in the home; ECE/ece = 2 years of more of centre based early childhood education prior to starting school/ not 2 years of ece; LOTE = Language other than English spoken

### Outcome 1: Good language (GL)

#### Necessity (see Table 5)

There were no conditions which fulfilled the set parameters to be considered necessary for GL and thus no condition was essential for GL to result at 5 years.

**Table 5:** Test for necessary conditions for Good (GL) and Poor Language (PL) outcomes

GL Conditions	Code	Consistency	Coverage
Good toddler development	D	0.83	0.92
2 or less children in the home	CH	0.76	0.96
No antenatal distress	AD	0.69	0.91
Good maternal responsivity in infancy & toddlerhood	RS	0.59	1
Maternal Education (left school 16 years or younger)	ME	0.52	0.83
Early Childhood Education: 2 or more years prior to school	CC	0.45	0.87
*Language Other Than English*	*LOTE*	*0.62*	*0.95*
*English speaking*	*English*	*0.38*	*0.73*
PL Conditions			
Poor maternal responsivity in infancy & or toddlerhood	rs	1	0.29
3+ children in the home	ch	0.80	0.36
Poor toddler development	d	0.60	0.38
Antenatal distress	ad	0.60	0.25
ECE: less than 2 years of centre based care prior to school	ece	0.60	0.16
Maternal education (left school 16 years or younger)	me	0.40	0.13
*Language Other Than English*	*LOTE*	*0.80*	*0.27*
*English speaking*	*English*	*0.20*	*0.03*

#### Sufficiency (see Table 4 and Fig. 3)

All conditions were kept for all analyses. There were 13 paths in the group leading to GL all of which had strong consistency [57] indicating the solution strongly relates to the outcome observed. It is notable that 5 of the 13 (Paths 3 & 10–13) paths represented no unique coverage, thus were present however not of high importance to the overall findings and have not been included in later analysis [56].

### Risk and protective conditions and paths to good language outcome

**Fig. 3:**
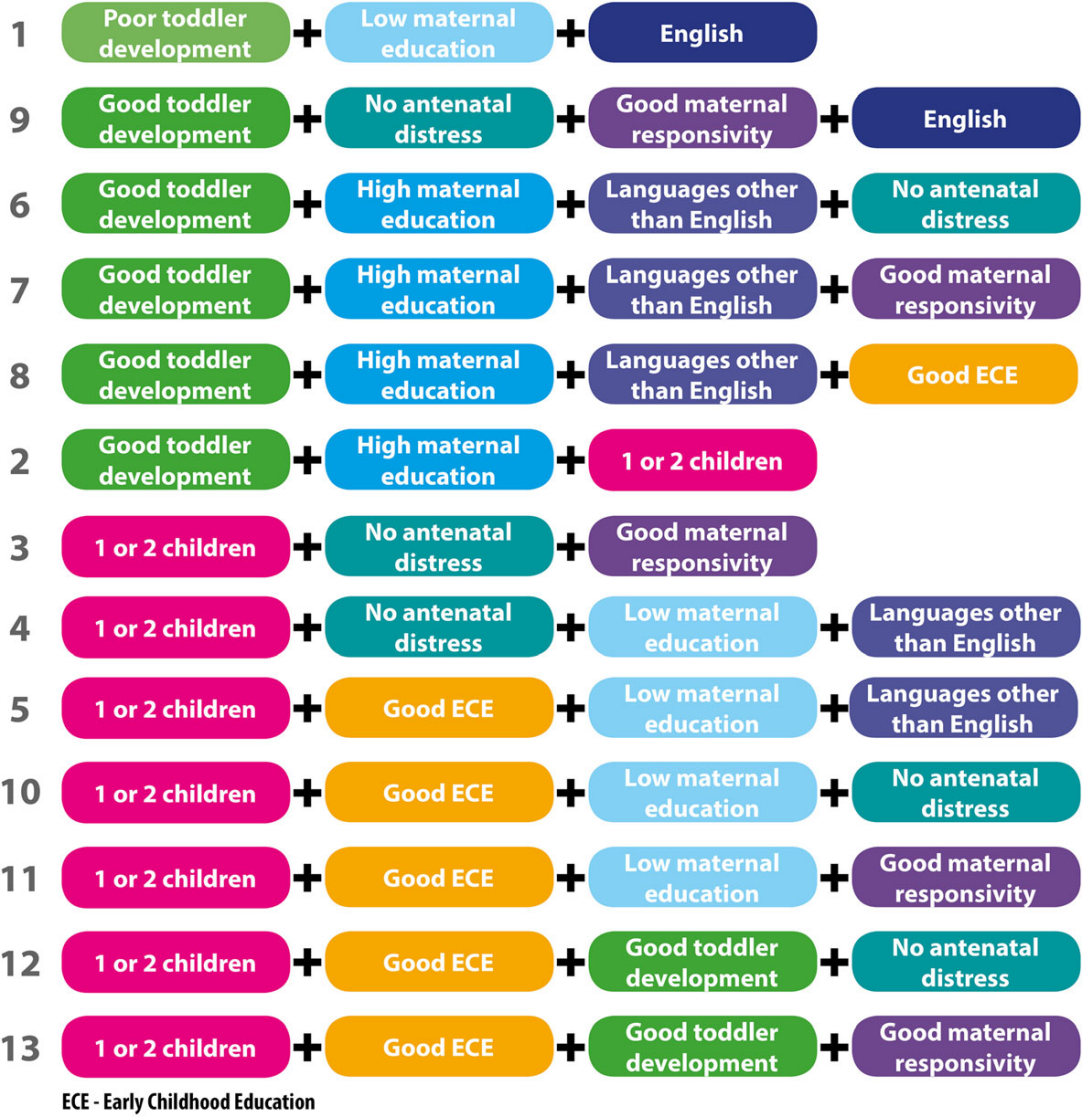
Paths to the good language outcome

The paths to GL were usually via the presence of no and minimal risk factors (see Fig. 3). There was one path of high risk to GL outcomes (Path 1), one of only two paths in which English speaking was sufficient*.* Eight of 13 paths were protective (Paths 2, 3, 6, 7, 8, 9, 12 & 13). That is, they contained only conditions in the protective mode. Overall, these paths consisted of all of the conditions in different combinations in protective mode: both manipulable (D, RS, ECE) and non manipulable (AD, CH, ME) conditions. All but one of these protective paths had good toddler development (D) as an influential condition (as indicated by the coverage score of 0.83), D was almost necessary for a Good Language outcome but not in all cases. Present in half of these paths was the combination D*ME (+*LOTE in one path).

There were four of 13 paths that were Mostly Protective (Paths 4, 5, 10 & 11). That is, they had one risk and this risk was always low maternal education. This risk was also always linked to having two or fewer children in the home (CH). Three of these four paths were also linked to two years or more of centre based early childhood education prior to starting school (ECE).

Most risk factors had no influence on any path to GL. These conditions were neither necessary nor sufficient: Having more than three children in the home (ch), not being responsive in infancy and toddlerhood (rs), having less than two years of early childhood education before starting school (ece) and being antenatally distressed (ad). That is, these risk factors were not influential in the outcome and were logically minimised out of all pathways to GL.

### Outcome 2: Poor language (PL)

#### Necessity (see Table 5)

For PL, poor maternal responsivity was a necessary condition, present in every path and every case however coverage was limited (consistency 1; coverage 0.29) due to the few cases with PL.

#### Sufficiency (see Table 4 and Fig. 4)

All conditions were kept for all analyses. The intermediate solution resulted in four paths in the group leading to poor language, all of which had acceptable consistency. This model had good solution coverage (1) and consistency (1). All paths provided some unique coverage.

**Fig. 4:**
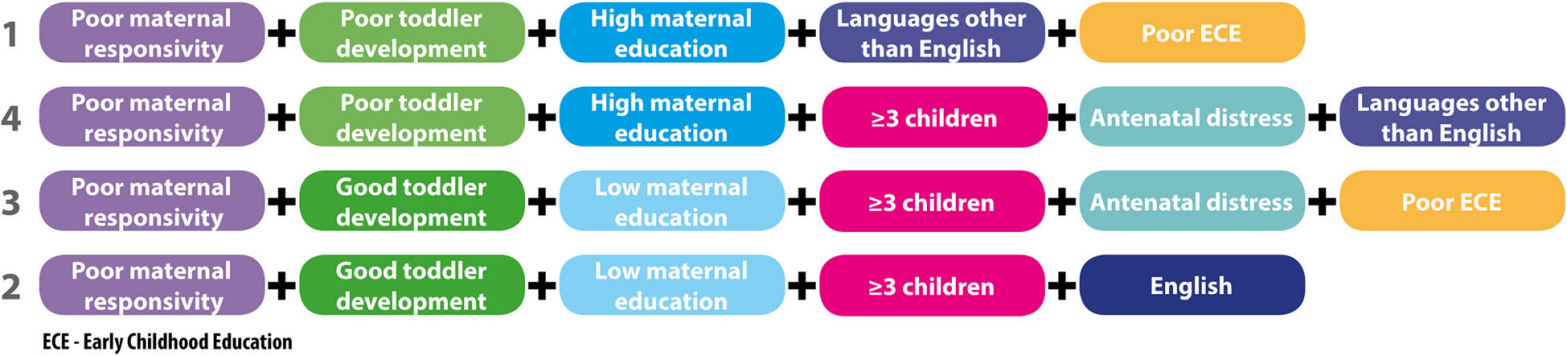
Paths to the poor language outcome

### Risk and protective conditions and paths to poor language

The paths to poor language were via the presence of risk factors and one protective factor (Fig. 4). The protective factor was either D or ME. Overall, these paths contained all of the conditions in different combinations in the risk mode: both manipulable (d, rs, ece) and non manipulable (ad, ch, me) conditions. As indicated by its necessary status, all paths contained poor maternal responsivity and three of the four also contained having 3 or more children in the home. The minimum number of risks conditions in each path was three. Both LOTE and English were influential in the paths to PL.

### Comparison of good and poor language paths

For this analysis only paths with unique coverage were included (GL Paths 3 & 10–13 were not considered). There was not complete symmetry in the paths to good and poor language outcomes, however there commonly was some when only the conditions were considered. Those conditions most consistently present for GL outcome (Table 6) were: good toddler development; higher maternal education; no antenatal distress; fewer children in the home and LOTE. For PL outcome most commonly present conditions were: good and poor toddler development; high and low maternal education; antenatal distress; poor maternal responsivity; three or more children in the home; non optimal amounts of centre based ECE prior to starting school. As evident above and in Table 6, there were a number of conditions with symmetry in GL versus PL outcome: the number of children in the home, early childhood education, maternal responsivity and antenatal distress. For all of these conditions the protective version was only present when GL resulted and the risk condition was only present with PL. For example good maternal responsivity (RS) was only ever present in the paths to GL and poor maternal responsivity (rs) was only ever present in the paths to PL. However, no other symmetry was present, the combinations of conditions in the paths were not the same. Maternal education and toddler development were two conditions that existed in both the risk and protective mode in both GL and PL outcomes. They also existed together in patterns: for GL, if development existed with maternal education they were both always in either the protective mode (5 paths) or both in the risk mode (1 path). In contrast for PL one was always protective and one always risk for example d*ME or D*me.

**Table 6:** Individual conditions presence influencing GL and PL outcomes for paths with unique coverage

Condition	GL: # of paths	% of GL paths	PL: # of paths	% of PL paths	Symmetry present
Toddler Development					
Good	5	56%	2	50%	No
Poor	1	11%	2	50%	No
Maternal Education					
Higher	4	44%	2	50%	No
Low	3	33%	2	50%	No
Antenatal distress					
None	4	44%	0	0%	Yes
present	0	0%	2	50%	Yes
Maternal Responsivity					
Good	3	33%	0	0%	Yes
Poor	0	0%	4	100%	Yes
Children in the Home					
Less	4	44%	0	0%	Yes
More	0	0%	3	75%	Yes
Early Childhood Education					
Optimal	2	22%	0	0%	Yes
Non optimal	0	0%	2	50%	Yes
LOTE	5	56%	2	50%	No
English speaking	1	11%	1	25%	No

## Discussion

This study used a novel mixed method, Qualitative Comparative Analysis (QCA) to explore the risk and protective pathways to good and poor language development in a group of at risk children. This method resulted in 13 different paths to good language (GL) and four to Poor Language (PL) outcomes. Multiple paths to the same outcome (equifinality) were present. That is, this empirical data demonstrates there are a variety of risk and protective pathways to both GL and PL outcomes. Also present was conjunctual causation, that is a variety of condition combinations to the outcomes were present. Overall, there was no one risk or protective factor that was necessary (present in all pathways) for good language outcomes. However, poor maternal responsivity in infancy and toddlerhood was necessary for poor language outcome at five years. Protective factors dominated the paths to GL with risk factors rarely being influential. In contrast, paths to PL outcomes always included poor maternal responsivity and a range of other risk factors. The model showed both causal symmetry and asymmetry, that is some conditions such as maternal responsivity, acted symmetrically. When mothers were responsive this was only influential to GL outcomes (it was protective) and when poor was only influential to PL outcomes (was a risk factor). Other conditions such as maternal education and toddler development functioned assymetrically and combined with other conditions to be influential in both good and poor language outcomes.

Currently we know that at risk children have a higher prevalence of PL than children not experiencing adversity [12]. The at risk children with GL in this study had factors present that appear to be protective against such poor language outcomes. Consistent with previous research, these children were in part predictable by their lack of influential risks and the presence of protective factors such as good toddler development, fewer children in the home, two years or more of early childhood education prior to starting school and no antenatal distress in the mother. Only five of 13 paths to GL included risk factors, four of which had one risk factor and this was always low maternal education (Paths 5, 6, 10 & 11). This finding provides some nuance about the role of maternal education in language acquisition. It is often found in large cohort studies that high maternal education is an influencing factor in GL outcomes [10, 46]. Our findings are no different, however they also explicate the context in which low maternal education can be related to GL outcomes for their child. As Harding, Morris (25) highlighted maternal education is a broad concept which represents a range of maternal skills and parenting practices. In this study, good language development occurred in children of mothers with low education *when combined* with having fewer children in the home and usually with the child having two years or more of early childhood education prior to starting school. Interestingly the language spoken in the home did not affect this finding – mothers of children with good language at five years spoke either English or another language (more commonly they were LOTE).

Consistent with previous work [47] the pathways for children with poor language (PL) were notable for many risk factors: specifically this study highlighted the importance of poor maternal responsivity in combination of other influential risk factors. Particularly evident in all cases of PL was the influential effect of poor maternal responsivity. In this study as in previous ones, children of less responsive mothers demonstrate more limited language than children of more responsive mothers [29, 63–65]. This study adds other key conditions that combine with poor responsivity to result in these poorer outcomes – in particular more children in the home, antenatal distress and or limited early childhood education. We assert, the compounding effect these conditions which result in less individualised and shaped responses to a child, impacts over time culminating in lower language at school entry. Similar to the pathways for GL, both high and low maternal education was associated with PL at five years. In addition, toddler development at three years, either good or poor, was associated with PL.

Toddler development emerged consistently as important and influential, particularly for GL outcomes. In all of the *no risk* paths to GL, good toddler development was influential in the outcome over and above other characteristics, reflective of previous findings [32]. As currently debated in the literature, children’s previous language and developmental outcomes are often good predictors of their later language outcomes but not always [4] . A large, longitudinal cohort study of children’s language development found 6% of children presenting with language difficulties at four years were not predicted by outcomes at two years and 14% of children with concerning development at two years had typical development at four years [10]. This study gives us more direction as to the combination of factors which result in the more unpredictable outcome. In Path 1 toddler development was poor and GL also resulted when combined with the influential effects of using a LOTE and low maternal education. Thus good development was not necessary for GL outcomes. As was the case for GL - both good and poor toddler development resulted in PL. It is possible this result may be explained by a missing condition which is hypothesised to influence the later emergence (in the early school years) of language concerns: genetic inheritance [66, 67]. Many children born to parents with poor language will also have difficulties: hereditability varies with age and testing method but reveal hereditability estimates of .24–.92, but as twin studies have shown both genes and environment may be involved and there is a complex relationship between these [68–73] . The evidence is still emerging, however it is thought ‘good’ genes may ‘protect’ against poorer environmental influences and ‘poor’ genes increase risk despite good environments and this may be evident (but untested) in these cases [74]. Future research requires family history of speech and language difficulties to be collected and included in models.

In QCA the cases of conflict (same pathway but different outcome) provide guidance as to where models may be deficient. There were four conflict cases in this data set Path 6 (cases 36 & 74) and Path 7 (cases 73 & 44) which were removed from the analysis. Their exclusion made minimal difference, however exploring these cases qualitatively is important for understanding how paths vary. The outcome for case 73 (PL at 5 years) may have been different to the paired case (44) due to the difference in English language exposure (there were few risks present in these cases). Though both were LOTE, case 44 reported speaking English as the main language to their child at 2 years of age. At the same age case 73 reported speaking a LOTE. This is consistent with Hoff’s [75] findings that, the more rich exposures to the dominant language the better the child will be in that language. For cases 36 and 74 a key point of difference was the behaviour score at three years, which was poor for the former and high for the latter. Although toddler behaviour was usually correlated with good development, this was one of the cases where it was not. Including more conditions such as toddler behaviour in analysis could have potentially resolved this conflict, and provided a further important path to PL but could not be supported by the sample size in this study.

### Policy and program implications

There are a number of policy and practice implications from our findings. Continued investigation of and investment in targeted treatments which impact on the responsivity of mothers early interactions with infants and toddlers, particularly targeting those mothers who are at greater risk and those with antenatal signs of distress is needed. As this and previous studies show, targeting maternal responsivity alone will not be enough to improve language outcomes, or reduce the incidence of language difficulties [76]. Rather treatments are needed that have the flexibility to target the range of risks present in this group, such as poor maternal responsivity, and parenting many children in the home, and/or child participation in high quality early childhood education programs. One such promising treatment may be sustained home visiting treatments starting antenatally and continuing until the child is two or three years old [77–79]. The findings also suggest that policies supporting implementation of long term early childhood education in areas of high disadvantage are helpful for children’s language development. We advocate that at risk children of mothers experiencing adversity require both very early public health interventions such as home visiting and then high quality, long term early childhood education to ameliorate the impacts of adversity.

### Limitations

There are a number of limitations in this study. This is case based work, aiming to determine causality within the sample and as such cannot be easily generalised to other populations, particularly as this study had a small sample and only eight cases had poor language. This number was smaller than could be expected in a group of at risk children based on the prevalence of poor language outcomes in the literature. This may have been impacted upon by the presence of initiatives focused on improving community level disadvantage in the study area at the time. Goldfeld, O’Connor (23) have developed a new model of disadvantage for children experiencing adversity, which includes aspects of neighbourhood environments not included in this study (e.g. neighbourhood liveability) that may have shed light on this finding. Additionally, this study population may have been socio economically relatively well resourced when compared to some other studies of children at risk. Although they lived in a low SES area, only 20% of the mothers were on welfare benefits. However, they were psychologically burdened with over 60% of the cohort presenting with either depression and or mental health difficulties and experiencing a range of significant adverse experiences, which have been shown to have impacts on children’s development.

The small sample also limited the diversity of conditions that could be included in the model. Choice of conditions was further limited by secondary analysis of existing data: other variables that are known to influence language acquisition, such as genetic bases or family history of language difficulties were not collected. The limitation in conditions explored is evidenced by the conflict cases discussed above. The decision to do this study as crisp set for clarity in this first exploration of language development with QCA may also have limited some nuance in the analysis.

## Future research

Future research of the pathways to good and poor language requires larger samples with purpose-designed/collected conditions. Continuing to use QCA is recommended for its ability to capture and elucidate the complexity of pathways to good and poor language, and hence support a more nuanced discussion about how to facilitate good language development and ameliorate poor language development in at risk children.

## Conclusion

This mixed methods study of language development in at risk children demonstrated there are varied pathways to both good and poor language outcomes. Most paths to good language involved protective factors, though not always. Similarly, but not symmetrically, paths to poor language more often involved risk factors. Key to poor language outcomes was poor maternal responsivity combined with other risk factors associated with poorer language development. Other conditions which differentiated the paths to good and poor language were the number of children in the home and toddler development. The complex pattern of factors associated with language outcomes suggests the need for complex interventions which can respond to these varied risk and protective patterns.

## Additional file

Additional file 1: Paths to language development in at risk children: a Qualitative Comparative Analysis (QCA). (PDF 1990 kb)

## Abbreviations

AD: Antenatal Distress
ANCTR: Australian New Zealand Clinical Trials Registry
Bayley MDI: Mental Development Index
CES-D: Center for Epidemiologic Studies Depression Scale
CH: The number of Children in the Home
D: Toddler Development
ECE: Early Childhood Education
EPDS: Edinburgh Post Natal Depression Scale
GL: Good Language
LOTE: Language Other Than English
MECSH: Maternal Early Childhood Sustained Home Visiting
PL: Poor Language
QCA: Qualitative Comparative Analysis
RCT: Randomised Control Trial
RS: Maternal Responsivity
SES: Socio Economic Status
VIQ: Verbal Intelligence Quotient
WPPSI-III: Wechsler Preschool and Primary Scale of Intelligence

## References

[1] Conti-Ramsden G, Durkin K, Simkin Z, Knox E (2009). Specific language impairment and school outcomes. I: identifying and explaining variability at the end of compulsory education. International Journal of Language & Communication Disorders.

[2] Johnson CJ, Beitchman JH, Brownlie E (2010). Twenty-year follow-up of children with and without speech-language impairments: family, educational, occupational, and quality of life outcomes. American Journal of Speech-Language Pathology.

[3] Schoon I, Parsons S, Rush R, Law J (2010). Childhood language skills and adult literacy: a 29-year follow-up study. Pediatrics.

[4] McKean C, Wraith D, Eadie P, Cook F, Mensah F, Reilly S (2017). Subgroups in language trajectories from 4 to 11 years: the nature and predictors of stable, improving and decreasing language trajectory groups. J Child Psychol Psychiatry.

[5] Norbury CF, Vamvakas G, Gooch D, Baird G, Charman T, Simonoff E (2017). Language growth in children with heterogeneous language disorders: a population study. J Child Psychol Psychiatry.

[6] Law J, Reilly S, Snow PC (2013). Child speech, language and communication need re-examined in a public health context: a new direction for the speech and language therapy profession. International Journal of Language & Communication Disorders.

[7] Collisson BA, Graham SA, Preston JL, Rose MS, McDonald S, Tough S (2016). Risk and protective factors for late talking: an epidemiologic investigation. J Pediatr.

[8] Le HN, Gold L, Mensah F, Eadie P, Bavin EL, Bretherton L (2017). Service utilisation and costs of language impairment in children: the early language in Victoria Australian population-based study. International journal of speech-language pathology.

[9] Eadie P, Conway L, Hallenstein B, Mensah F, McKean C, Reilly S. Quality of life in children with developmental language disorder. Int J Lang Commun Disord. 2018;53(4):799-810. 10.1111/1460-6984.12385 29577529

[10] Reilly S, Wake M, Ukoumunne OC, Bavin E, Prior M, Cini E (2010). Predicting language outcomes at 4 years of age: findings from early language in Victoria study. Pediatrics.

[11] Law J, Todd L, Clark J, Mroz M, Carr J (2013). Early language delays in the UK.

[12] Law J, Charlton J, Dockrell J, Gascoigne M, McKean C, Theakston A (2017). Early Language Development: Needs, provision, and intervention for preschool children from socio-economically disadvantage backgrounds. Institute of Education-London.

[13] Clegg J, Ansorge L, Stackhouse J, Donlan C (2012). Developmental communication impairments in adults: outcomes and life experiences of adults and their parents. Language, Speech, and Hearing Services in Schools..

[14] Brownlie EB, Graham E, Bao L, Koyama E, Beitchman JH (2017). Language disorder and retrospectively reported sexual abuse of girls: severity and disclosure. J Child Psychol Psychiatry.

[15] Beitchman JH, Jiang H, Koyama E, Johnson CJ, Escobar M, Atkinson L (2008). Models and determinants of vocabulary growth from kindergarten to adulthood. J Child Psychol Psychiatry.

[16] Brownlie E, Beitchman JH, Escobar M, Young A, Atkinson L, Johnson C (2004). Early language impairment and young adult delinquent and aggressive behavior. J Abnorm Child Psychol.

[17] Beitchman JH, Wilson B, Douglas L, Young A, Adlaf E (2001). Substance use disorders in young adults with and without LD: predictive and concurrent relationships. J Learn Disabil.

[18] Beitchman JH, Wilson B, Johnson CJ, Atkinson L, Young A, Adlaf E (2001). Fourteen-year follow-up of speech/language-impaired and control children: psychiatric outcome. J Am Acad Child Adolesc Psychiatry.

[19] Snowling MJ, Adams JW, Bishop D, Stothard SE (2001). Educational attainments of school leavers with a preschool history of speech-language impairments. Int J Lang Commun Disord.

[20] Hughes N, Chitsabesan P, Bryan K, Borschmann R, Swain N, Lennox C (2017). Language impairment and comorbid vulnerabilities among young people in custody. J Child Psychol Psychiatry.

[21] Conti-Ramsden G, Durkin K (2012). Postschool educational and employment experiences of young people with specific language impairment. Language, Speech, and Hearing Services in Schools.

[22] Caspi A, Houts RM, Belsky DW, Harrington H, Hogan S, Ramrakha S (2016). Childhood forecasting of a small segment of the population with large economic burden. Nat Hum Behav.

[23] Goldfeld S, O’Connor M, Cloney D, Gray S, Redmond G, Badland H (2018). Understanding child disadvantage from a social determinants perspective. J Epidemiol Community Health.

[24] Bronfenbrenner U. Making human beings human: bioecological perspectives on human development. Thousand Oaks: Sage; 2005.

[25] Harding JF, Morris PA, Hughes D (2015). The relationship between maternal education and children’s academic outcomes: a theoretical framework. J Marriage Fam.

[26] Frech A, Kimbro RT (2011). Maternal mental health, neighborhood characteristics, and time investments in children. J Marriage Fam.

[27] Baydar N, Kuntay AC, Yagmurlu B, Aydemir N, Cankaya D, Goksen F (2014). "it takes a village" to support the vocabulary development of children with multiple risk factors. Dev Psychol.

[28] Tough SC, Siever JE, Leew S, Johnston DW, Benzies K, Clark D (2008). Maternal mental health predicts risk of developmental problems at 3 years of age: follow up of a community based trial. BMC pregnancy and childbirth.

[29] Smith J, Levickis P, Eadie T, Bretherton L, Conway L, Goldfeld S. Concurrent associations between maternal behaviours and infant communication within a cohort of women and their infants experiencing adversity. Int J Speech Lang Pathol. 2018;20(5):516-27. 10.1080/17549507.2017.1329458 28682122

[30] Reilly S, Wake M, Bavin EL, Prior M, Williams J, Bretherton L (2007). Predicting language at 2 years of age: a prospective community study. Pediatrics.

[31] Barre N, Morgan A, Doyle LW, Anderson PJ (2011). Language abilities in children who were very preterm and/or very low birth weight: a meta-analysis. J Pediatr.

[32] Durand VN, Loe IM, Yeatman JD, Feldman HM (2013). Effects of early language, speech, and cognition on later reading: a mediation analysis. Front Psychol.

[33] Law J, Rush R, Parsons S, Schoon I (2013). The relationship between gender, receptive vocabulary, and literacy from school entry through to adulthood. International Journal of Speech-Language Pathology..

[34] Farrant BM, Zubrick S (2013). R. Parent–child book reading across early childhood and child vocabulary in the early school years: findings from the longitudinal study of Australian children. First Lang.

[35] McKean C, Mensah FK, Eadie P, Bavin EL, Bretherton L, Cini E (2015). Levers for language growth: characteristics and predictors of language trajectories between 4 and 7 years. PLoS One.

[36] Harrison L, McLeod S (2010). Risk and protective factors associated with speech and language impairment in a nationally representative sample of 4- to 5-year-old children. Journal of Speech, Language, and Hearing Research.

[37] Choudhury N, Benasich AA (2003). A family aggregation study: the influence of family history and other risk factors on language development. Journal of Speech, Language, and Hearing Research..

[38] Short K, Eadie P, Descallar J, Comino E, Kemp L (2017). Longitudinal vocabulary development in Australian urban aboriginal children: protective and risk factors. Child Care Health Dev.

[39] Tayler C (2016). The E4Kids study: Assessing the effectiveness of Australian early childhood education and care programs: overview of findings at 2016.

[40] Farkas G, Beron K (2004). The detailed age trajectory of oral vocabulary knowledge: differences by class and race. Soc Sci Res.

[41] Nelson KE, Welsh JA, Trup EMV, Greenberg MT (2011). Language delays of impoverished preschool children in relation to early academic and emotion recognition skills. First Lang.

[42] Rice ML, Hoffman L (2015). Predicting vocabulary growth in children with and without specific language impairment: a longitudinal study from 2; 6 to 21 years of age. Journal of Speech, Language, and Hearing Research..

[43] Law J, Dennis JA, Charlton JJV. Speech and language therapy interventions for children with primary speech and/or language disorders. Cochrane Database Syst Rev. 2017, Issue 1. Art. No.: CD012490. 10.1002/14651858.CD012490.

[44] Reilly S, Bavin EL, Bretherton L, Conway L, Eadie P, Cini E (2009). The early language in Victoria study (ELVS): a prospective, longitudinal study of communication skills and expressive vocabulary development at 8, 12 and 24 months. International Journal of Speech-Language Pathology.

[45] Zubrick SR, Taylor CL, Rice ML, Slegers DW (2007). Late language emergence at 24 months: an epidemiological study of prevalence, predictors, and covariates. Journal of Speech, Language, and Hearing Research..

[46] Taylor CL, Christensen D, Lawrence D, Mitrou F, Zubrick SR (2013). Risk factors for children's receptive vocabulary development from four to eight years in the longitudinal study of Australian children. PLoS One.

[47] Christensen D, Taylor CL, Zubrick SR (2017). Patterns of multiple risk exposures for low receptive vocabulary growth 4-8 years in the longitudinal study of Australian children. PLoS One.

[48] Conti-Ramsden G, Durkin K (2015). What factors influence language impairment considering resilience as well as risk. Folia Phoniatrica et Logopaedica.

[49] Johnson SB, Riis JL, Noble KG (2016). State of the art review: poverty and the developing brain. Pediatrics.

[50] Brofenbrenner U, Morris P, Damn W, Lerner RM (1998). Theoretical models of human development. Handbook of child psychology.

[51] Assmussen K, Feinstein L, Martin J, Chowdry H (2016). Foundations for life: what works to support parent child interaction in the early years.

[52] Blackman T, Wistow J, Byrne D (2013). Using qualitative comparative analysis to understand complex policy problems. Evaluation..

[53] Kemp L, Harris E, McMahon C, Matthey S, Vimpani G, Anderson T (2008). Miller early childhood sustained home-visiting (MECSH) trial: design, method and sample description. BMC Public Health.

[54] Cox JL, Holden JM, Sagovsky R (1987). Detection of postnatal depression: development of the 10-item Edinburgh postnatal depression scale. Br J Psychiatry.

[55] Wechsler D (2002). Wechsler preschool and primary scale of intelligence (WPPSI-III). Third edition ed.

[56] Schneider CQ, Wagemann C. Set-theoretic methods for the social sciences: a guide to qualitative comparative analysis. Cambridge: Cambridge University Press; 2012.

[57] Ragin C (2008). Redesigning social inquiry: set relations in social research.

[58] Rihoux B, Ragin C (2009). Configurational comparative methods: qualitative comparative analysis (QCA) and related techniques.

[59] Caldwell BM, Bradley RH (2003). Home inventory administration manual: University of Arkansas for Medical Sciences.

[60] NICHD ECCRN (1999). Child care and mother-child interaction in the first 3 years of life. Dev Psychol.

[61] Quine WV (1952). The problem of simplifying truth functions. Am Math Mon.

[62] Quine WV (1955). A way to simplify truth functions. Am Math Mon.

[63] Smith J, Eadie T, Levickis P, Bretherton L, Goldfeld S. Predictive validity of verbal and non-verbal communication and mother–child turn-taking at 12 months on language outcomes at 24 and 36 months in a cohort of infants experiencing adversity: a preliminary study. International Journal of Language & Communication Disorders. 2018. 10.1111/1460-6984.12408 29999217

[64] Landry SH, Smith KE, Swank PR, Zucker T, Crawford AD, Solari EF (2012). The effects of a responsive parenting intervention on parent-child interactions during shared book reading. Dev Psychol.

[65] Hudson S, Levickis P, Down K, Nicholls R, Wake M (2015). Maternal responsiveness predicts child language at ages 3 and 4 in a community-based sample of slow-to-talk toddlers. International journal of language & communication disorders..

[66] Zambrana IM, Pons F, Eadie P, Ystrom E (2014). Trajectories of language delay from age 3 to 5: persistence, recovery and late onset. International Journal of Language & Communication Disorders..

[67] Snowling MJ, Duff FJ, Nash HM, Hulme C (2016). Language profiles and literacy outcomes of children with resolving, emerging, or persisting language impairments. J Child Psychol Psychiatry.

[68] Dale P, Simonoff E, Bishop D, Eley T, Oliver B, Price T (1998). Genetic influence on language delay in two-year-old children. Nat Neurosci.

[69] Rice ML, Zubrick SR, Taylor CL, Gayán J, Bontempo DE (2014). Late language emergence in 24-month-old twins: heritable and increased risk for late language emergence in twins. Journal of Speech, Language, and Hearing Research..

[70] Rice ML, Zubrick SR, Taylor CL, Hoffman L, Gayán J (2018). Longitudinal study of language and speech of twins at 4 and 6 years: twinning effects decrease, zygosity effects disappear, and heritability increases. Journal of Speech, Language, and Hearing Research.

[71] Hayiou-Thomas ME, Dale PS, Plomin R (2012). The etiology of variation in language skills changes with development: a longitudinal twin study of language from 2 to 12 years. Dev Sci.

[72] Dale PS, Dionne G, Eley TC, Plomin R (2000). Lexical and grammatical development: a behavioural genetic perspective. Journal of child language.

[73] Newbury DF, Fisher SE, Monaco AP (2010). Recent advances in the genetics of language impairment. Genome medicine.

[74] Rice ML (2013). Language growth and genetics of specific language impairment. International Journal of Speech-Language Pathology..

[75] Hoff E (2013). Interpreting the early language trajectories of children from low SES and language minority homes: implications for closing achievement gaps. Dev Psychol.

[76] Wake M, Tobin S, Girolametto L, Ukoumunne OC, Gold L, Levickis P (2011). Outcomes of population based language promotion for slow to talk toddlers at ages 2 and 3 years: Let’s learn language cluster randomised controlled trial. Br Med J.

[77] Landry SH, Smith KE, Swank PR, Guttentag C (2008). A responsive parenting intervention: the optimal timing across early childhood for impacting maternal behaviors and child outcomes. Dev Psychol.

[78] Olds DL, Kitzman H, Knudtson MD, Anson E, Smith JA, Cole R (2014). Effect of home visiting by nurses on maternal and child mortality: results of a 2-decade follow-up of a randomized clinical trial. JAMA Pediatr.

[79] Olds DL, Kitzman H, Cole R, Robinson J, Sidora K, Luckey DW (2004). Effects of nurse home-visiting on maternal life course and child development: age 6 follow-up results of a randomized trial. Pediatrics..

[80] Bayley N (1993). Bayley scales of infant development.

[81] Kemp L, Harris E, McMahon C, Matthey S, Vimpani G, Anderson T (2011). Child and family outcomes of a long-term nurse home visitation programme: a randomised controlled trial. Arch Dis Child.

