# Supplementary material for: Paths to language development in at risk children: a qualitative comparative analysis (QCA)
Source: BMC Pediatr. 2019 Apr 5;19:94. doi: 10.1186/s12887-019-1449-z (PMC6449893; doi:10.1186/s12887-019-1449-z)
Supplement: Supplementary file 1 — Paths to language development in at risk children: a Qualitative Comparative Analysis (QCA). (PDF 1990 kb) [file 12887_2019_1449_MOESM1_ESM.pdf]

Additional file 1: Paths to language development in at risk children: a Qualitative Comparative Analysis (QCA)

Short, Eadie & Kemp.

Contents

Additional file 1: Paths to language development in at risk children: a Qualitative Comparative Analysis (QCA) 1
Short, Eadie & Kemp. 1
MECSH Qualitative Comparative Analysis (QCA): Outcome and All Conditions Explained 2
1. Aim of the study / Research question 2
2. What is QCA? 2
3. Some methodological information 3
Measures and Data Collection: 3
4. Language Outcome Measure 3
4.1 WPPSI-III 3
4.2 Teacher Perception of Language Ability 4
2.3 The Language Outcome Criteria 4
5. The Conditions 6
5.1 Child Gender 6
5.2 Child's preschool development 6
5.3 Preschool Behaviour 7
5.4 Maternal Education 8
5.5 Maternal Antenatal Distress 8
5.6 Maternal Chronic Distress 9
5.7 Maternal Responsivity 11
5.8 Number of children in the home 12
5.9 Language Other than English (LOTE) 13
5.10 Family Origin 14
5.11 SES over time 15
5.12 Consistently Read to 3 Times a Week 17
5.13 Consistent optimal centre based early childhood education (ECE) 18
6. Calibrated Data Set 21
7. QCA Truth Table 23
8. Complex and Parsimonious Solutions without conflicts 24
4.1 Good Language Outcome 24
4.2 Poor Language Outcome 25
References 27

MECSH Qualitative Comparative Analysis (QCA): Outcome and All Conditions Explained

1. Aim of the study / Research question

What risk and protective factors impact on communication at 5 years old in a group of at risk children who are experiencing adversity?

2. What is QCA?

QCA is a mixed method standing between qualitative and quantitative methodologies and has been employed to help answer complex health policy questions. Boolean logic is used to investigate 3 key phenomena and further our knowledge of what causes an outcome: conjunctual causation, equifinality and causal asymmetry.

- *Conjunctual causation:* specific *combinations* of causes lead to a specified outcome. For example in language acquisition research low maternal education is often related to lower language outcomes in children, however there are always children of low educated mothers that have good language development. Thus, this condition is too blunt on its own to use as a risk determining the children who require preventative therapies. Cumulative risk models to determine who requires preventative interventions are called for (McKean et al., 2017) and investigations of conjunctual causation help determine the combinations of risk and protective conditions to place in these risk models. QCA uses empirical cases to investigate the theoretically driven variables (if any) which combine to result in an outcome. Thus our example requires us to ask, what other variables combine with low maternal education to result in good or poor language? One possibility could be that good language results when mothers of low education have a male child who has received good quality early childhood education (ECE).
- Equifinality refers to the multiple paths that lead to an outcome. In our example good language at 5 years may result from low maternal education + being male + having good quality ECE. However, an equally effective path to good language may be high maternal education + speaking a language other than English + good toddler development. This method may help move us closer to understanding the multiple combinations of risk and protective factors which result in and good and poor language outcomes. In turn, understanding these complex paths may help us develop more effective interventions.
- *Causal asymmetry* is an important methodological task of QCA. Causal asymmetry assumes desirable and undesirable outcomes are not the result of paths that are the direct opposite of each other. For example, from the above, just because low maternal education + being male + good ECE led to good language outcomes does not mean that high maternal education + being female + having poor ECE necessarily leads to poor language outcomes. The paths are not symmetrical. Similarly if we just think about the conditions, a condition such as having good quality ECE may be necessary for good language outcomes, but poor quality ECE may not be necessary for poor language.

The two mathematical concepts of necessity and sufficiency are investigated in QCA and are essential to understanding results. Each variable, called a condition in QCA, may be necessary or sufficient (or neither) for an outcome. For a condition to be necessary it must need to be present for the outcome to come about. For example, if all children who were read to 3 times a week or more in toddlerhood had good language at 5 years this condition would be *necessary* to good language outcomes. Necessity gives clear instructions for intervention. However, few conditions are usually necessary. More common is that conditions are *sufficient.* That is, when a condition is combined with one or more conditions it is causal for the outcome. In our example above, although low maternal education is not necessary for good language,

it is sufficient when combined with other conditions (being male and good quality ECE). Examination of the sufficient condition combinations will help develop more targeted language interventions for children experiencing adversity.

3. Some methodological information

Outlined in sections 2 & 3 is the creation of the Language Outcome measure and the 13 conditions. The data used, where it was sourced from and how calibration (cut points) were determined; how missing data was dealt with.

Participants: In the original RCT design, 41 participants received treatment and 41 comparison. This additional document outlines the 41 comparison participants used in this study.

Measures and Data Collection:

Table 1: Ages and tools for data collection:

**Child Age Data Collected**	**Tool**	**Condition Used For**
Antenatal Recruitment	Recruitment survey	Maternal Education; Number of children in the home; SES; Family Origin; Antenatal Distress
Birth	Hospital Records	Gender
12 & 24 months	HOME	Maternal responsivity
18months - 33months	Bayleys MDI	Preschool Development; Preschool Behaviour
	NICHD coded parent child play interactions	Maternal Responsivity
30-72 months	telephone survey every 6 months	Read to three times a week; Early Childhood Education; Chronic Maternal Distress
End Term 1 Kindergarten (cohort mean age = 64 months; sd=4.1 months)	WPPSI - VIQ	Language Outcome (for regression and QCA)
	Teacher perception scale	QCA Language Outcome

4. Language Outcome Measure

In an effort to represent functional as well as standardised test performance, end Language status, was determined by a combined criteria utilising a standardised test and teacher perception of child skill in Kindergarten. Both administered in the second term of the child's first year of formal schooling (Kindergarten). The standardised tool was the Wechsler Preschool and Primary Scale of Intelligence III (WPPSI-III) Australian Edition (Wechsler, 2002), specifically the verbal subscale. Added to this was the teachers assessment of the child's receptive and expressive language ability. Each of these tools is discussed separately below and then results combined to create the outcome.

4.1 WPPSI-III

Every child (N=41) who had a WPPSI is included in the cohort. Children completed a WPPSI in term 2 of their first year of school. The core subtests were completed for all children, thus a verbal (VIQ), performance (PIQ) and full subscales (FSIQ) were derived for all children. The verbal subscale (VIQ: Vocabulary, information and word reasoning subtests) was used.

- ### *Cut point and coding for this measure*

If a child's Standard Scores (SS) fell 1SD below (SS=84 or lower) in the VIQ they were placed in the Poor Language (PL) group. This is a standard cut point to consider a child's language of possible concern.

- ### *Result for this measure*

Total group mean age when test was conducted was 64.41 months (SD 4.1). Verbal Score mean score = 103.39 (SD 14.65), range= 78-130.

Good (GL) Vs Poor Language (PL)

4 (9.9%) of the 41 participants fulfil criteria for PL on the WPPSI, they had a mean SS of 80 (SD 2.5) (range= 78-83) and the GL group had a mean score of 105.92 (SD13.1), range= 85-130.

4.2 Teacher Perception of Language Ability

Teacher perception was measured on a likert scale completed by the teacher in term 2 of Kindergarten. Teachers answered 2 questions related to the child's language skills: "*Please think about the skills and competencies of the study child as described in the next statements. Rate how this child has compared with other children of a similar age, over the past few months. Expressive language skills (eg. Using language effectively, ability to communicate) and Receptive Language (eg. Understanding, interpreting and listening). Choice of 4 responses: 1. More competent than others 2. As competent as others 3. Less competent than others 4. Much less competent.*

These were reverse scored and the totals added together, so each child received a rating (possible range 2-8) for their language skill. The higher the rating the higher the teachers perception of the child's skill.

Children's teachers completed these rating scales on their students. Overall response rate of 88% (n=36). The teacher perception rating and WPPSI VIQ were moderately correlated across the sample (r=0.410, p=0.013).

Any child that received a (reverse) total score of 4 or less was considered PL. In this method, 5 of 36 (14%) were classed as PL. There is no possibility that a child considered above peers in either expressive or receptive language could be included in the group. There was a possibility that children who were considered same as peers in one of the realms could be included (this was so for case 64: normal receptive & expressive sig below peers, however WPPSI SS=78, in the PL range), so deemed appropriately placed in the PL group. *Note: There are 5 cases with missing teacher perception data.*

Table 2: Scores on the teacher perception scales divided by Good & Poor Language as indicated by teacher perception cut point

	GL[$]		PL[#]		P value
	**n**	**Mean (SD)**	**n**	**Mean (SD)**	
TOTAL	31	6.58 (1.025)	5	4.0 (0.00)	>0.000
Expressive		3.35 (0.551)		1.80 (0.447)	0.000
Receptive		3.23 (0.617)		2.20 (0.447)	0.001

[$]GL = Good Language [#]PL = Poor Language

2.3 The Language Outcome Criteria

Using the above, 1 (2%) student was detected as PL in both WIPPSI and teacher perception. 28 students were detected as GL in both methods (68%). Thus 70% of students there was agreement in the 2 methods. 4 students (10%) were considered PL by the teachers but had GL according to the WPPSI (false negatives) and 3 (7%) students were considered TL by the teachers but not by the WPPSI (false positives). There were 4 (10%) students who were classed as GL on the WPPSI and there was no teacher perception score for them

Table 3: Teacher perception and WPPSI grouped PL and GL

		WIPPSI		
		**GL$**	**PL#**	**TOTAL**
**teacher perception**	**GL$**	29	3	32
	**PL#**	4	1	5
**TOTAL**		**33**	**4**	**37**

$GL = Good Language #PL = Poor Language; Note 4 students with missing teacher perception data

Thus the following criteria was set: students were considered PL if they fulfilled PL criteria in either measure. Thus there are 8 children of PL (19.5% of the sample).

Table 4: Language Outcome (WPPSI verbal subscale SS and teacher perception score) divided by Good and Poor Language

	GL$		PL#		P value
	**n**	**Mean (SD)**	**n**	**Mean (SD)**	
TOTAL WIPPSI	33	107.12 (13.0)	8	88.0 (10.73)	< 0.000
TOTAL teacher perception	29	6.57 (1.3)	8	5 (1.5)	0.002

$GL = Good Language #PL = Poor Language

Table 5: Outcome coding for crisp set

Outcome	Code	Definition	Truth Table Code	n
**Good Language**	GL	Child scored Standard Score 85 or more on WPPSI and scored 5 or more on teacher perception scale.	1	33
**Poor Language**	PL	Child scored Standard Score 84 or less on WPPSI and / or scored 4 or less on teacher perception scale.	0	8

Managing the Missing data: There is missing data in the teacher perception scores (cases) (Case no's: 13, 20, 55, 66). There is no missing data in the WPPSI's.

Crisp Set: WIPPSI was given precedence. If only WPPSI was present, 0 or 1 coded on the WPPSI result. This led to all cases being coded 1 (Good language) as all cases with missing teacher perception data had WIPPSI's above 85.

5. The Conditions

The following conditions were examined as possible factors of influence on the MECSH cohort participants language development:

Table 6: The conditions and their code

	Brofenbenner's Ecological Systems Theory	Condition	Code for the condition
**1.**	Child:	Gender	G
**2.**		Preschool Development (at 32 months)	D
**3.**		Behaviour at 3 years	B
**4.**	Maternal:	Education	ME
**5.**		Distress antenatal	AD
**6.**		Chronic Distress	CD
**7.**		Maternal Responsivity	RS
**8.**	Environment / Family:	Number of children in the home	CH
**9.**		LOTE (Language Other Than English)	LOTE/ English
**10.**		Family Origin	FO
**11.**		SES (Socio Economic Status: Housing & Income)	SES
**12.**		Read to 3 Times a Week	RD
**13.**		Optimal centre based Early Childhood Education (ECE)	ECE

In crisp set QCA, each condition (factor) is coded as 1 or 0. 1= Presence of the condition and 0 = Absence of the condition. In set theory, presence of the condition is *in the set* and absence of the condition is *out of the set*.

5.1 Child Gender

Rationale: Gender has been long thought a predictor of language skill. This has been shown in the early years of language acquisition though its influence has been shown to wash out over time (Law, Rush, Parsons, & Schoon, 2013; Short, Eadie, Descallar, Comino, & Kemp, 2017).

This data was collected from the child's hospital records at birth. 100% response rate. There are more females (n=22) than males (n=19) in the cohort.

Coding for crisp set:

Table 7: Gender crisp set coding

Condition	Code	Total n	n GL	n PL	Truth Table Code
**Male**	M	19	16	3	0
**Female**	F	22	17	5	1

Managing the Missing data: There is no missing data in this set.

5.2 Child's preschool development

Rationale: Children's earlier developmental level is a known strong predictor of later language skill (Durand, Loe, Yeatman, & Feldman, 2013).

The children were tested on the Bayley scales of infant development (Bayley, 1993) by trained psychologists in the clinic. Age at testing: 32 months (2:8yrs; SD 4.3 months) range 17 -43 months. (1:5-

3:9), median age 32 months (note 1 outlier tested at 17months all others from 27 months). See table 8 for the means and SD of age and SS of Bayley scores the whole sample and divided by language status at 5 years.

Table 8: Preschool Development

	N	Age months (SD)	SS mean (SD)	Range	N participants typical Preschool development	N participants atypical preschool development **(1SD below)**
TOTAL	40	32 (4.4)	98.48 (14.96)	67-122	32 (80%)	8 (20%)
GL	32	31.53 (4.31)	100.25 (14.73)	67-122	27(84%)	5 (16%)
PL	8	34.13 (4.36)	91.38 (14.67)	69-116	5(63%)	3 (37%)

Good preschool development was indicted by SS 1 SD from mean and above (SS 85 and above).

Coding for crisp set:

Table 9: Preschool Development crisp set coding

Condition	Code	Descriptor	n	Truth Table Code	SS mean (SD)
**Preschool Development**	D	Development within 1SD & above	32	1	103.75 (11.01)
	d	Development 1SD below mean (SS <84)	8	0	77.38 (6.16)

Managing the Missing data: There was 1 cases of missing data (case no: 10). This resulted in case 10 being removed from all QCA analysis.

5.3 Preschool Behaviour

Rationale: Behaviour and attention has been consistently linked to language acquisition in a complex manner. It is unproven the direction of influence language on behaviour or behaviour on language, though it is likely to be bi -directional with likely key influencing moderators being social disadvantage, gender and cognition (Law, Plunkett, & Stringer, 2012).

The behaviour score (percentile rank) from the Bayley scales of infant development (Bayley, 1993) was utilised. This was obtained at the same assessment as the MDI (Preschool development measure above). The Bayleys Behaviour score is based on observation throughout the Bayleys test administration. The examiner marks three factors each element on a 5 point rating scale: Emotional Regulation (task persistence, attention, frustration, activity level and adaptability); Motor Quality (muscle tone fine and gross motor movement), and Orientation/Engagement (interest, initiation interaction and response to positive affect). Messigner, et al (2010) found behaviour scores on the Bayleys II predicted later cognitive scores. Good preschooler behaviour was indicted by PR 26 and above.

Ages at testing same as for preschool behaviour: 32 months (2:8yrs) (SD 4.3 months) range 17 -43 months. (1:5-3:9), median age 32 months. (note 1 outlier tested at 17months all others from 27 months). One child was not tested (case no. 10)

Table 10: Preschool Behaviour

	N	Age (SD)	PR mean (SD)	Range	N participants typical Preschool behaviour	N participants atypical preschool behaviour **(25th PR & below)**
TOTAL	40	32 (4.4)	61.55 (34.18)	0-99	30 (75%)	10 (25%)
GL	32	31.53 (4.3)	65.25 (33.78)	4-99	25 (78%)	7 (22%)
PL	8	34.13 (4.36)	46.75 (33.77)	0-95	5 (63%)	3 (47%)

Coding for crisp set:
Cut point was as outlined by the manual PR 25 (Bayley, 1993).

Table 11: Preschool Behaviour coding for crisp set

Condition	Code	Descriptor	n	Truth Table Code	SS mean (SD)	Range
**Preschool Behaviour**	B	Behaviour within 1SD & above (PR 26 and above)	30	1	78.00 (20.94)	29-99
	b	Behaviour 1SD below mean (PR <25)	10	0	12.20 (6.48)	0-23

**significant difference between the two groups as expected (p=0.000)

Managing the Missing data: There was 1 case of missing data (case no: 10) and this case was removed from the QCA analysis.

5.4 Maternal Education

Rationale: Every year a mother stays at school has impacts on child outcomes (Harding, Morris, & Hughes, 2015). Age mothers left school was collected at the recruitment survey, as was the highest level of education attained by the time of the child's birth. 38 of 41 (93%) of participants completed the age left school.

Coding for crisp set:
The sample was then dichotomously coded for crisp set:

- 1= left school at 17 years or older (n= 20)
- 0= Left school at 16 years or less (n= 18)

Managing the Missing data: There were 3 cases with missing data from age left school (cases 11,60,63) and one of these who also has no level of education information (Case no: 11). This case was dropped from the QCA analysis as no information could be found to complete this information. The 2 cases with missing data from age left school (cases 60,63), but have highest level of education and are: case 60- bachelors degree; case 63- high school. Case 60 was placed in the set (higher education) due to the attainment of a Bachelors degree. Case 11 was unclear and excluded from further analysis in the QCA.

Table 12: Maternal education coding for crisp set

Condition	Code	Descriptor	n	Truth Table Code
**Maternal Education**	ME	Left school at or after 17 years	20	1
	me	Left school at or before 16 years	18	0
**Missing**			2	

5.5 Maternal Antenatal Distress

Rationale: Mothers mental state has been shown to influence language development over time (Baydar et al., 2014; Tough et al., 2008) and maternal antenatal distress has been related to poor language and developmental outcomes in longitudinal studies (Talge et al., 2007; Van den Bergh, Mulder, Mennes, & Glover, 2005). In the earlier MECSH RCT maternal antenatal distress was shown to influence child development outcomes (Kemp et al, 2011). Mothers in this study received routine psychosocial screen and the Edinburgh Postnatal Depression Scale (EPDS) in the hospital when they presented for their first antenatal appointment (usually under 20 weeks pregnant). All mothers in this cohort who were found to have depression and or mental health difficulties also scored as distressed in this cohort.

Data was collected from the recruitment survey and antenatal hospital records. The EPDS score was used as the measure for maternal mental status – called antenatal maternal distress.

Coding for crisp set:
Consistent with clinical practice guidelines of the time a score of 10 was used as the cut point for clinical concern and entry to the study (Kemp et al., 2011).
The sample was then dichotomously coded for crisp set:

- 1= 9 or under EDPS at recruitment (n=29)
- 0= 10 or over EPDS at recruitment (n=12)

Table 13: Maternal antenatal distress crisp set coding

Condition	Code	n	Mean EPDS Score (SD)	Truth Table Code
**Maternal Antenatal Distress**	AD	29	4.26 (1.5)	1
	ad	12	11.8 (2.7)	0

Managing the Missing data: There were 2 cases of missing raw data in Maternal Antenatal Distress (Case no's: 11, 26). These were manually filled from hospital records – which only stated if the mothers EPDS was over or under 10. Both of these scored under 10 and were placed in the set=1.

5.6 Maternal Chronic Distress

Rationale: Long term effects on child language and cognitive development of chronic post partum depression have been reported (Grace, Evindar, & Stewart, 2003; Murray et al., 2010); and significant impact of maternal stress over time on development (Shonkoff et al., 2011).

Data was collected from 5 surveys of maternal distress/ depression anxiety collected over the course of the study (see table 14 below for collection times and tools). Survey interviews were conducted over the phone and in the home. Two tools have been used the Edinburgh Postnatal Depression Scale(EPDS)and The Center for Epidemiologic Studies Depression Scale (CES-D).

TABLE 14: Chronic Distress tools and times assessed

	Childs Age	Assessment period	Tool used
**1**	12 months	12 month survey/ home visit	EPDS
**2**	average age 17 months	1st DA	CES-D
**3**	24 months	24 month survey/ home visit	EPDS
**4**	average age 33 months	2nd DA	CES-D
**5**	48 months	48 month survey	CES-D

Cut points for the tools:

- the Edinburgh Postnatal Depression Scale(EPDS) I used 10 as the cut point (Kemp et al., 2011)

- The Center for Epidemiologic Studies Depression Scale (CES-D) a cut point of 16 as outlined in the MESCH coding guideline

Each set of data was then dichotomously coded:

- 1 (less risk) for EDS 9 or under or CES-D 15 or under
- 0 (more risk) EDS 10 or over or CES-D 16 or over

Two thirds (66%) of the sample have had all or most (4 or 5 of 5) post birth distress assessments. Analysis of the number of assessments conducted postnatally (max possible 5) divided by the number of times a participant scored as distressed (see Table 15). 56% of the participants never reported distress. 24 (28%) participants reported distress some but less than half of the time they were asked. 14% (6 participants) reported distress more than half of the time postnatally.

Table 15: % of assessments conducted mothers reported distress

% of times distress reported	N participants	% of participants
**0**	23	56%
**20-40**	11	28%
**60**	1	2%
**100**	5	12%

Looking at times mothers reported distress (see table 16), there was a range from 18- 35% of the sample reported as distressed at any one time point and there seemed to be a peak time at 17 months (average) - though this was also the time point of the least assessments (26 of 41 had this assessment).

Table 16: Maternal Distress outcomes by each time point and overall

<table>
<tr><th></th><th>Outcome</th><th>N</th><th>Participants (%)</th><th>mean score (SD)</th></tr>
<tr><td rowspan="2">EPDS at 12 months</td><td>Normal</td><td rowspan="2">28</td><td>23</td><td rowspan="2">4.9 (5.2)</td></tr>
<tr><td>Borderline/ Abnormal</td><td>5 (18%)</td></tr>
<tr><td rowspan="2">CESD at 17 months</td><td>Normal</td><td rowspan="2">26</td><td>17</td><td rowspan="2">15.73 (5.3)</td></tr>
<tr><td>Borderline/ Abnormal</td><td>9 (35%)</td></tr>
<tr><td rowspan="2">EPDS at 24 months</td><td>Normal</td><td rowspan="2">31</td><td>24</td><td rowspan="2">6.8 (6.1)</td></tr>
<tr><td>Borderline/ Abnormal</td><td>7 (23%)</td></tr>
<tr><td rowspan="2">CESD at 33 months</td><td>Normal</td><td rowspan="2">40</td><td>33</td><td rowspan="2">8.3 (12.1)</td></tr>
<tr><td>Borderline/ Abnormal</td><td>7 (18%)</td></tr>
<tr><td rowspan="2">CESD at 48mth survey</td><td>Normal</td><td rowspan="2">36</td><td>28</td><td rowspan="2">9.6 (12.2)</td></tr>
<tr><td>Borderline/ Abnormal</td><td>8 (22%)</td></tr>
<tr><td rowspan="2">Chronic Distress</td><td>not distressed 50% of times or more</td><td rowspan="2">41</td><td>35</td><td></td></tr>
<tr><td>Distressed 50% of times or more</td><td>6 (15%)</td><td></td></tr>
</table>

Using a conservative cut point it was determined that 50% of times asked mother presented as distressed would be considered chronic distress. There was a concern that for those with only 2 or 3 assessments (see

table 17) chronicity would not be shown – however the cases that had only 2 – 3 assessments were not distressed (every time) or distressed every time.

Table 17: Maternal Distress Assessments

Number of Ax's Postnatally	n	%	No. of cases distressed	% of cases distressed
**2**	3	7%	1	33%
**3**	11	27%	1	9%
**4**	13	32%	1	8%
**5**	14	34%	3	21%
**total**	41		6	15%

Managing the Missing data: The method of determining the outcome meant there was no case with missing data, despite not every case having every assessment.

Coding for crisp set:
The sample was then dichotomously coded for crisp set:

- 1= distress present less than 50% of the time (n=35)
- 0= 50% or more of the times distress was measured the mother presented with a score on either the EPDS or the CESD in the range of concern (n= 6)

Table 18: Maternal antenatal distress coding for crisp set

Condition	Code	n	Truth Table Code
**Maternal Antenatal Distress**	AD	35	1
	Ad	6	0

5.7 Maternal Responsivity

Rationale: Maternal responsivity has consistently been shown to impact on the language development of children experiencing adversity in the short and long term. Both the quality and quantity of the interaction have been shown to impact, though recently there is a strong argument for the quality of the responsiveness rather than just the amount of talk. Responsivity has been measured in various ways in these studies. (Bornstein, Tamis-LeMonda, Hahn, & Haynes, 2008; Evans, Boxhill, & Pinkava, 2008; Hirsh-Pasek et al., 2015; Levickis, Reilly, Girolametto, Ukoumunne, & Wake, 2014; Milgrom, Westley, & Gemmill, 2004; Paavola, Kunnari, & Moilanen, 2005; Pearson et al., 2012; Smith, Landry, & Swank, 2006; Tamis-LeMonda, Kuchirko, & Song, 2014; H. B. Taylor, Anthony, Aghara, Smith, & Landry, 2008).

Quality of Maternal responsivity under 2 years Quality of Maternal responsivity was operationalized combining a home based analysis using the Responsivity score (i.e. appropriateness and timeliness of responsiveness to child bids for attention) of the Infant Toddler HOME Score (Caldwell & Bradley, 2003) and rating of maternal child interaction play sample analysis conducted in the clinic. The 11 point Responsivity score of the HOME assessment was elicited in a semi-structured interview in the participants home when the child was around 2 years (mean age 24 months, SD 4.7) (n=40). There was 1 child who was not assessed on the HOME. The play samples were elicited at mean age 20.5 months (SD= 8.0months) (n= 38). There were three children who were not assessed. The play samples were rated using the NICHD mother child interaction coding system (NICHD, 1999) on 4 maternal dimensions related to language stimulation: sensitivity to the child (i.e. supportive of child's goals and desires/not intrusive), stimulation, detachment & intrusiveness. Each dimension was rated on a 4-point scale (from 4 very characteristic to 1 not characteristic at all) detachment and intrusiveness both reverse scored. The play sample scores and the HOME

responsivity rating were then added to together to create a measure of language stimulation. A good quality language environment was indicted by higher score with the range possible being 4-27. (This method modelled on (Belsky et al., 2007).

In the standardisation of the HOME Infant toddler: mean for the Responsivity subscale was 8 (SD=2.2) and median 9 (Caldwell & Bradley, 2003). If either the NICHD or the HOME score fell below the sample mean score for that measure (HOME Mean = 9.4 cut point = 9; NICHD mean= 9.9, cut point = 10), the case was considered of concern.

Managing the missing data:

There are 4 participants with one missing responsivity score (either NICHD or HOME- no case was missing both) (Cases 1, 10, 24 & 57). 3 of these cases with missing data were already coded because the score they did have fell in the concerning range (Cases 1, 10 & 57). For case 24, it was missing the NICHD score and had a HOME score of 9. The score was imputed from other data in the set. In the sample there are 15 cases with a HOME score of 9 and an NICHD score. Mean NICHD score of these cases= 10 (9.73) SD 3.8 (range 5-15). Thus the imputed score = 10. This means this case is *in the set* for both NICHD and HOME and classed in the set (1) for the crisp set analysis.

This resulted in the following calibration:

Coding for crisp set:

Table 19: Maternal Responsivity coding for crisp set

Condition	Code		Mean of 2 scores combined	SD	Range of 2 scores combined	n	Truth Table Code
**Good Maternal Responsivity**	RS	Scores for HOME and NICHD are above the sample mean (9 or above on HOME and 10 or above on NICHD)	22.95	2.2	8-20	21	1
**Poor Maternal Responsivity**	rs	Scores for HOME and /or NICHD are below the sample mean (8 or below on HOME or 9 or below on NICHD)	16.10	2.6	19-27	20	0

5.8 Number of children in the home

Rationale: The number of children in the home has been found to influence vocabulary acquisition in a local similar cohort of children experiencing adversity (Short et al., 2017) and in various other Australian and International cohorts (Harrison & McLeod, 2010; Tomblin, Hardy, & Hein, 1991; Zubrick, Taylor, Rice, & Slegers, 2007).

This data was collected at recruitment to the study via survey. As outlined below in table 20, 32% (n=13) of the cohort were the first child in the family. 66% had 2 or less children at the study child's birth. The number of children in families ranged from 1-7 children (including the study child). Mean 2.27 (SD 1.3) children in the families.

Table 20: Number of children in the home at study child's birth.

Number of children in the home	no of cases	% of participants
**1**	13	31.7%
**2**	14	34.1%
**3**	8	19.5%
**4**	4	9.8%
**5**	1	2.4%

**6**	0	0
**7**	1	2.4%
**total**	41	

Managing the Missing data: There were 2 missing data points in the number of children in the home (2 cases) (Case no's: 11, 15). Information in the participant study file was found indicating the number of siblings quoted from the mother. This data has been used.

Coding for crisp set:

Table 21: Number of children in the home coding for crisp set

Condition	Code		n	Mean # children (SD)	Truth Table Code
**Less children**	CH	2 children or less in the home	27	1.52 (0.5)	1
**More children**	ch	3 children (including the study child) or more in the home	14	3.7 (1.1)	0

5.9 Language Other than English (LOTE)

Rationale: Using a language other than the dominant language of the country (in this case English) in which the child lives has been related to heightened risk *of* lower early language acquisition, often with a dramatic improvement over time (Christensen, Taylor, & Zubrick, 2017; Christensen, Zubrick, Lawrence, Mitrou, & Taylor, 2014; McKean et al., 2017; C. L. Taylor, Christensen, Lawrence, Mitrou, & Zubrick, 2013). This may be related to testing being conducted in the language in which they are not dominant, in this case English, but may be also be related to lower quality models and exposure in the countries dominant language, and the amount the is exposed to and child talks in each of their languages(Hoff, 2018; Ribot, Hoff, & Burridge, 2018). Minority families, often not speaking the dominant language are overrepresented in low SES cohorts, and this is thought to confound the relationship of SES and language outcome. However, it has been shown this is not the case (Hoff, 2006).

Though there are many features of being Culturally and Linguistically Diverse (CALD) that may impact on a child's outcome, and this variable was originally called CALD. However, it was considered that for this study the most crucial element that may affect outcome is the language the child uses / is exposed to, particularly as children were only tested in English. It is recognised the importance and benefit of speaking more than one language.

The languages used in the home were collected by parent report at the developmental assessments. They reported languages used at home and primary language used with the child.

Every family reported speaking English sometimes. 83% (n=34) of families reported English was the primary language spoken at home and 80% (n=33) reported English was the primary language spoken with the child. However, this is not representative of the language use in the homes.

19 (46%) families reported speaking a language other than English in the home and 22 (54%) of families spoke only English. There were 12 languages (or groups of languages*) other than English spoken. Arabic was the most common language spoken other than English. *all dialects of a language have been reported under the main language e.g. Hakka a Chinese dialect and Piedmontese an Italian dialect.

Table 22: Languages spoken in the home

An Aboriginal Language	1	2%	German	1	2%
Arabic	7	17%	Greek	1	2%
Assyrian	1	2%	Hindi	1	2%
Cambodian	1	2%	Italian	1	2%
Chinese	2	4%	Samoan	3	7%
English only	22	56%	Spanish	2	4%

Managing the Missing data: There was one case reported only English at one time, but an Aboriginal Language (which language was not outlined by the parent) and German at another (case no:69). This case was coded as using a language other than English at home.

Coding for crisp set:

Table 23:LOTE coding for crisp set

Condition	Code		n	Truth Table Code
English only	English	English only spoken	22	1
LOTE	LOTE	Language other than English spoken in the home	19	0

5.10 Family Origin

Rationale: When working with the SES, maternal education and languages spoken data, there became apparent a link to recently being a migrant. This may be reflected by Australian migrant policy at the time which encouraged skilled migrant immigration, thus the first generation migrants though economically not well off, had a different maternal education level and over time higher SES. The eventual issue with this condition may have been the correlation with other conditions.

There is family of origin data on all 41 cases.

Mothers are Aboriginal (13% of sample)

- 5 of 41 cases (Case No's: 13, 25,35,38,69) mothers identified as Aboriginal (12%). 4/5 mothers and maternal grandparents were born in Australia. There is one case no. 69 Aboriginal case not born in Australia - this cases mother (study child's grandparent) was born in Australia, the study child's mother identifies as Aboriginal, documents speaking an Aboriginal Language at home. She was born elsewhere and migrated (back) to Australia when a young child. She has been considered Aboriginal not migrant (despite being born OS). 1 case did not respond to the question of Aboriginality and was born in Australia.

Mothers are Non Aboriginal, Non recent Migrant (29% of sample)

- 12 of 41 (29%) cases were not Aboriginal and mothers and grandparents were born in Australia these are considered 2 generation or more non migrant. Of these non- migrant 12 cases, 2 had a language other than English being spoken in the home Samoan in both cases– presumably the language of the father/ his family. 3/41 Don't have data on grandparents (Cases 11, 60,63)- All of these mothers were born in Australia have been categorised in this section as non migrant (cases: 1, 3, 9, 10, 11 28, 60, 63, 66, 68, 71, 74).

Mothers are Second Generation Migrants (24% of sample)

- 10/41 grandparents but not mothers were born overseas (the study child's mother is a Second generation migrant) thus are second generation migrants. 5 speak a language other than English in the home (Cases: 6, 18, 21, 24, 26, 31, 36, 55, 72, 73).

Mothers are First Generation Migrants (34% of sample)

- 34% (14/ 41) both mothers and grandparents were not born in Australia – first generation migrants (Case no:20, 30, 41, 44,45,46,47, 51 ,52,54,57, 61 ,64, 79)
- Of these 33 cases the mothers migration regions were:

Table 24: Mothers migration region

Region	Countries	n	% of the whole cohort
**Middle East**	Iraq, Lebanon	5	36%
**South America**	Argentina, Uruguay	2	14%
**Pacific**	East Timor, Fiji, New Zealand, Samoa	4	28%
**Asia**	Hong Kong	1	7%
**Europe (including UK)**	England	2	14%

Table 25:Mothers migration Ages

Age arrived	Number	% of all
**Under 5 years**	5	36%
**6-12 years**	5	36%
**13-18 years**	1	7%
**19- 25**	3	21%
**TOTAL**	14	

Managing the Missing data: There were 5 participants with missing grandparent data – outlined above how these have been dealt with. All cases classified.

Coding for crisp set:

Table 26: Coding for Family Origin Crisp Set

Condition	Code		n	Truth Table Code
**FAMILY ORIGIN**	FO	First generation migrant	14	1
	fo	Not first generation migrant (Aboriginal, second and longer generation migrant)	27	0

5.11 SES over time

Rationale: There is a significant body of research demonstrating a relationship between language acquisition and SES broadly. However there is some argument that it could be the other related elements (represented here in other conditions) which are the real influences of language outcome rather than SES per se. (Johnson, Riis, & Noble, 2016; Melvin et al., 2017; Noble et al., 2015; Piccolo, Merz, He, Sowell, & Noble, 2016; Ursache & Noble, 2016)

Note this cohort were recruited from a low SES area, so there may be limited meaningful differences between them. Data was collected from 2 surveys in an attempt to get a longitudinal measure of SES. Income data was not directly available however employment and household accommodation were. These 2 constructs (self reported by parents in 2 interviews) were used combined as a measure of SES (). Survey interviews were conducted (over the phone) at:

- Recruitment to the study (mostly completed antenatally with a few completing these post natally)
- 48 months (Child 4 years)

The same survey questions were used in the 2 interviews:

2. What would best describe your family's main source of income (Only one answer)

A. Full time employment (coded 1)
B. Part time employment (coded 2)
C. Benefit (eg. sickness/unemployment) (coded 3)
D. Pension (coded 4)
E. Other ______________________(coded 5)

Each participants response on both surveys was then dichotomously coded:

- 1 (protective) for employed (full or part time) and
- 0 (risk) benefit pension or other

5. Which best describes your household's accommodation?

1. Fully Owned (coded 1)
2. Being Purchased (coded 2)
3. Being Purchased under rent/buy scheme (coded 3)
4. Rent Public (coded 4)
5. Rent Private (coded 5)
6. Living rent free (coded 6)
7. Other____________________________(coded 7)

Each participants response on both surveys was then dichotomously coded:

- 1 (protective) for home ownership (purchased or being purchased) and
- 0 (risk) non home ownership (rent, living rent free or other)

Table 27: SES Income and employment over time

		Recruitment survey	48 month survey
Main Income Source	Employed part or full time	32 (78%)	25 (61%)
	Not employed (pension benefit or other)	8 (20%)	11 (27%)
	Missing	1 (2.4%)	5 (12%)
Household Accommodation	Own or purchasing home	22 (54%)	21 (51%)
	rent or other	18 (44%)	15 (37%)
	Missing	1 (2.4%)	5 (12%)

Note there were 6 participants that had incomplete data 1 did not complete the recruitment survey and 5 did not complete the 48 month survey. All participants had at least one survey complete.

Each question response was coded as outlined above so participants with both surveys complete had 4 possible coded responses each of which I will consider an SES risk or protective factor. Those with only 1 survey complete had 2 possible coded responses. Once each questions response was coded, the % of responses falling to the risk criteria were collated. Those with 50% risk or more considered lower SES.

- 1 (protective) = those with less than 50% risk across the two time points n=21.

- 0 (risk)= those with risk indicated 50% or more of the time n= 20

Table 28: SES percentage of participants income and employment risk over time

#participants %income & employment risk		% of cohort	In or out of the set
**0%**	12	29%	1
**25%**	9	22%	1
**50%**	16	39%	0
**75%**	0	-	0
**100%**	4	10%	0

Managing the Missing data: See above for missing survey information. The method of determining the outcome meant there was no case with missing data, despite not every case having every assessment.

Coding for crisp set:

Table 29: Coding for SES crisp set

Condition	Code		n	Truth Table Code
**Higher SES**	SES	49% or less housing and income low	21	1
**lower SES**	ses	50% of the time or more housing and income low	20	0

5.12 Consistently Read to 3 Times a Week

Rationale: Being read to consistently has been demonstrated to be related to current and future language and literacy skills (Kim, Im, & Kwon, 2015; McKean et al., 2015; Sénéchal et al., 2017; C. L. Taylor et al., 2013).

Being read to more than three times a week was collected via parent report, three times between 12 months and 3 months prior to school entry (4-5 yrs). The first two data collection times were 12 and 24 months when the HOME (Caldwell & Bradley, 2003) was administered in the home via a semi structured interview. Question 42 on the HOME requires a yes/ no response to "does the parent report reading stories to child at least 3 times weekly". This question was each coded 1 (yes) or 0 (no). At 3 months prior to starting school a survey was completed by parents over the phone/ at a home visit. The following data was obtained:

Table 30: Read to data converted to a binomial

Parent report following question: In the past week have you or someone in your family read to your child from a book?	Code	Converted to a binomial*
**child not read to**	0	0
**child read to on 1-2 days**	1	0
**Child read to on 3-5 days**	2	1
**Child read to everyday (6-7days)**	3	1

*This data was converted to a binomial 0= children read to less than 3 times a week and 1= children read to 3 times or more a week

The following data was available on all 41 cases:

Table 31: Read to data over time

Read to 3 times a week or more						
	12 month		24 months		Prior to School	
**N completed**	29	70%	34	83%	40	98%
**No**	7	17%	12	29%	13	32%
**Yes**	22	54%	22	54%	27	66%
**Missing**	12	29%	7	17%	1	2%

The majority 23 (57%) of the cases have data for all 3 time points and all others except one have 2 of the 3 data points. 1 case (Case no. 1) has no data, and this has resulted in this case being removed from all QCA analysis. All other cases missing data: 17 of 41 (41%) are missing data for only one time point: 11 missing the data from the 12 month survey and 6 missing data from the 24 month survey. Except for case 1, there is no missing data

It was determined that if it was ever recorded that the child had not been read to three times a week or more, they were at risk for poorer exposure to positive reading experiences.

To investigate the impact of having data for 2 not 3 assessment times, we compared the rates of being read to every time vs sometimes vs never comparing those with all assessments and those without (see table 32). Of the 57% (23) with all data present 13 (57%) were coded as "risk" for being read to ie had at least one time point where they were not read to 3 times a week. In comparison to only the cases with only 2 assessment points, only 35% (6 cases) were classified as "risk". Thus, there may be over identification of consistent readers.

Table 32: Missing data analysis for "read to" condition.

	Read to always	Not read to 33- 66%	Not read to 100%
**Cases with data at all time points**	10	10	3
	43%	43%	13%
Cases with 2 assessments only	11	5	1
	64%	29%	7%

Managing the Missing data: There is missing data point case no. 1 and this case will be removed from the QCA.

Coding for crisp set:

Table 33: Read to coding for crisp set

Condition	Code		n	Truth Table Code
**Consistently Read to**	RD	**Presence of Good Consistent Reading** Were read to 3 times a week on every occasion asked	21	1
**Not Consistently Read to**	rd	**Absence of Good Consistent Reading:** were not read to 3 times a week or more on more than one occasion	19	0

5.13 Consistent optimal centre based early childhood education (ECE)

Rationale: Three key markers of ECE have been shown to impact on later outcomes including language: quality of interactions between educators and children, the amount of childcare and the age at which ECE begins (Arcos Holzinger & Biddle, 2015; Sylva et al., 2004; Taggart, Sylva, Melhuish, Sammons, & Siraj, 2015; Tayler, 2016). We had no measures of quality of care available, however did have age when started and for how long they received care prior to starting school

Parents reported information about ECE at 9 possible times from 12 months to just prior to starting school. Each participant completed at minimum 2 of these surveys, though the mean completion was 6 surveys Mode was 7 surveys (Range:2-8; SD1.6). They reported data on the: Use, type and name of Early Childhood Education; hours in Early Childhood Education; age child started child care; number of kids in the preschool/ child care (parent estimate) from 2 ½ years – just prior to school entry. These were completed as outlined in table 34.

Table 34: Surveys completed for ECE data.

Child Age Survey completed	12 months	24 months	30 months	36 month (3 yrs)	42 month	48 month (4 yrs)	54 month	60 month (5 yrs) [#]	Prior to School (PtS)*[#]
**N completed**	25	33	3	21	25	36	31	28	40
**% of sample**	61%	80%	7%	51%	61%	88%	76%	68%	98%

*average age of completion: 59 months (4.1) Range: 52-70mths

[#]Note: The 60 month survey was conducted at the same time as the Prior to School Survey for 10 cases (24% of cases). Thus these 10 children only had data for possibly 8 surveys (Cases: 6, 18, 44,45, 54,60,63, 66, 74, 79).

Type of care the child was attending was coded into 2 options as outlined in table 35.

Table 35: Type of Care

Type of Care	N	Av. Age starting school (SD)	Definition
**Centre based care ECE**	35	61 months (3.9)	On at least two surveys had to have centre based ECE for at least one day week (and had attended for at least one year)
**Parental and Informal care: family day care; other family members, nannies.**	6	64 months (4.2)	The parent reported some type of informal care such as Family day care or occasional care; or parent care only (never more than 2 surveys stating the child received centre based ECE)

Length of time in centre based ECE: This was established for the 35 children who had received 12 months or more of centre based ECE. This was the time between starting ECE and school entry. The age of starting centre based care was determined, as was the age of starting school and a simple equation was utilised to establish amount of time in care (age started school – age started centre based care). To further

analyse length of time in care, the centre based data was coded into 2 categories – 23+ months of centre based care, and those with 12-23 months of care.

Table 36: Age started centre based ECE and length of time in centre based care by care type

Type of Care	N	Av. Age (SD) started care (range) in months	P value	Average months (SD) in care before starting school	P value	Av.age months (SD) started school	P value
**12-22 months in centre based ECE**	16	44.18 (6.4)	<0.000	15.5 (4.1)	<0.000	60.9 (4.6)	.425
**23+ months in centre based ECE**	19	35.58 (3.7)		26.3 (4.5)		61.89 (3.3)	

46% (19) of the cohort received centre based care for almost 2 years (23 months) or more. Another 39% received centre based care for 12-23 months. 15% (6) did not receive at least 12 months of centre based care.

Coding of Crisp Set:

Table 37: Coding of ECE for crisp set

Condition	Code	Definition	n	Truth Table Code
**Early Childhood Education**	CC	Presence of 23+ months of centre based ECE	19	1
	cc	Absence of any centre based ECE and 22 months or less of centre based ECE	22	0

6. Calibrated Data Set

Case No.	Language Outcome	G	D	B	ME	AD	CD	RS	CH	LOTE	FO	ses	RD	ECE
1	1	0	0	0	0	0	1	0	1	0	0	0		1
3	1	0	1	1	0	1	1	0	1	1	0	0	1	1
6	1	0	1	1	0	1	1	0	1	1	0	1	0	1
9	1	0	1	0	0	1	1	1	1	1	0	1	1	1
10	1	1		0	1	1	1	0	1	1	0	1	1	1
11	0	0	1	1		0	0	1	0	1	0	0	1	0
13	1	1	0	0	0	1	1	1	1	1	0	0	0	0
18	1	1	1	1	0	1	1	1	0	1	0	0	0	0
20	1	1	1	1	1	1	1	0	1	1	1	1	0	1
21	1	1	1	1	1	1	0	1	1	1	0	0	0	0
24	1	0	1	1	1	0	1	1	0	0	0	1	1	0
25	1	1	1	1	0	0	1	1	1	1	0	0	1	1
26	1	0	1	1	1	1	1	0	1	1	0	1	1	0
28	1	1	1	0	1	1	1	1	0	1	0	0	0	0
30	1	0	1	1	1	0	1	1	1	0	1	1	0	1
31	0	1	1	1	0	0	1	0	0	0	0	1	0	0
35	1	0	0	1	0	0	1	0	0	1	0	0	1	0
36	0	1	1	0	0	1	1	1	0	0	0	0	1	0
38	0	0	1	1	0	1	1	0	0	1	0	0	0	1
41	1	1	1	1	1	0	1	0	0	0	1	1	1	1
44	1	1	1	1	1	1	1	0	1	0	1	1	1	1
45	0	1	0	0	1	0	1	0	0	0	1	1	0	0
46	1	1	1	1	0	1	1	1	1	0	1	1	0	0
47	1	0	0	0	0	1	0	0	1	0	1	1	1	0
51	1	1	1	1	1	0	1	0	1	0	1	1	1	0
52	0	1	0	0	1	0	0	0	0	0	1	0	0	1
54	1	0	1	1	1	1	1	0	1	0	1	1	0	0
55	1	0	1	1	1	1	1	1	1	1	0	1	1	1
57	1	1	1	1	1	0	1	0	1	1	1	0	0	0
60	1	1	1	1	1	1	1	1	1	1	0	1	1	1
61	1	0	1	0	1	1	1	0	0	0	1	1	0	0
63	1	1	1	1		1	1	1	1	1	0	0	1	1
64	0	0	0	1	1	1	1	0	1	0	1	0	0	0
66	1	0	1	1	0	1	1	1	0	1	0	0	1	1
68	1	0	1	1	1	1	0	1	1	1	0	0	0	0
69	1	1	1	1	1	1	1	1	1	0	0	1	1	1

71	1	0	1	1	0	1	1	1	1	1	0	1	0	0
72	1	1	1	1	0	0	1	1	1	1	0	0	1	1
73	0	1	1	1	1	1	1	0	1	0	0	0	1	1
74	1	1	1	1	0	1	0	1	0	0	0	0	0	0
79	1	0	0	0	0	1	1	1	1	0	1	1	1	0

Note cases with missing data were removed from further analysis.

7. QCA Truth Table

Path	D$	ME^	AD*	RS#	CH˜	ECE¥	English / LOTE∞	Language Status	Raw consistency	# of cases in path	Case/s
1	1	0	0	0	0	0	0	0	0	1	31
2	0	1	0	0	0	0	0	0	0	1	45
3	0	1	1	0	1	0	0	0	0	1	64
4	0	1	0	0	0	1	0	0	0	1	52
5	1	0	1	0	0	1	1	0	0	1	38
6	1	0	1	1	0	0	0	1	0.5	2	36, 74
7	1	1	1	0	1	1	0	1	0.5	2	44, 73
8	1	1	1	1	1	0	1	1	1	2	21, 68
9	1	0	1	0	1	1	1	1	1	2	3,6
10	1	0	0	1	1	1	1	1	1	2	25,72
11	1	1	1	1	1	1	1	1	1	2	55,60
12	1	1	1	0	0	0	0	1	1	1	61
13	1	1	0	1	0	0	0	1	1	1	24
14	1	1	0	0	1	0	0	1	1	1	51
15	0	0	1	0	1	0	0	1	1	1	47
16	1	1	1	0	1	0	0	1	1	1	54
17	0	0	1	1	1	0	0	1	1	1	79
18	1	0	1	1	1	0	0	1	1	1	46
19	0	0	0	0	0	0	1	1	1	1	35
20	1	0	1	1	0	0	1	1	1	1	18
21	1	1	1	1	0	0	1	1	1	1	28
22	1	1	0	0	1	0	1	1	1	1	57
23	1	1	1	0	1	0	1	1	1	1	26
24	0	0	1	1	1	0	1	1	1	1	13
25	1	0	1	1	1	0	1	1	1	1	71
26	1	1	0	0	0	1	0	1	1	1	41
27	0	0	0	0	1	1	0	1	1	1	11
28	1	1	0	1	1	1	0	1	1	1	30
29	1	1	1	1	1	1	0	1	1	1	69
30	1	0	1	1	0	1	1	1	1	1	66
31	1	1	1	0	1	1	1	1	1	1	20
32	1	0	1	1	1	1	1	1	1	1	9
Rows 33 – 128: Logical Remainders											

D$ = toddler development; ME^= Maternal: education; AD= antenatal distress; RS#=Responsivity in infancy and toddlerhood; CH˜=Number of children in the home; ECE¥=2 years of more of centre based early childhood education prior to starting school; LOTE∞= Language other than English spoken.

Note paths 6 and 7 contravened the parameters set for consistency and were removed from further analysis.

8. Complex and Parsimonious Solutions without conflicts

4.1 Good Language Outcome

4.1.1 GL Necessary Conditions

Conditions tested:	Consistency	Coverage
**PreschoolDevelopment**	0.827586	0.923077
**~PreschoolDevelopment**	0.172414	0.625000
**MaternalEducation**	0.517241	0.833333
**~MaternalEducation**	0.482759	0.875000
**Maternaldistressantenatally**	0.689655	0.909091
**~Maternaldistressantenatally**	0.310345	0.750000
**Responsivity**	0.586207	1.000000
**~Responsivity**	0.413793	0.705882
**Childreninhome**	0.758621	0.956522
**~Childreninhome**	0.241379	0.636364
**English**	0.620690	0.947368
**~LOTE**	0.379310	0.733333
**Early Childhood Education**	0.448276	0.866667
**~Early Childhood Education**	0.551724	0.842105

4.1.2 GL Tests of Sufficiency: Complex Solution

****
TRUTH TABLE ANALYSIS
****

File: F:/PhD/Data/MECSH Papers/Trial 7 QCA comparison only no conflicts.csv
Model: GoodLanguageOutcome = f(Preschool Development, Maternal Education, Maternal distress antenatally, Responsivity, Children in home, LOTE, Early Childhood Education)
Algorithm: Quine-McCluskey

Frequency cut off: 1; Consistency cut off: 1
Cases with greater than 0.5 membership in term:

Path	Formula	Coverage		Consistency	Cases
		**Raw**	**Unique**		
**1**	D*ME*rs*CH*ece	0.137931	0.103448	1	26 , 51 , 54 , 57
**2**	me*AD*RS*CH*ece	0.137931	0.0689655	1	13 , 46 , 71 , 79
**3**	D*AD*RS*English*ece	0.172414	0.103448	1	18, 21, 28, 68, 71
**4**	D*me*AD*RS*English	0.137931	0.0344828	1	9 , 18 , 66 , 71
**5**	D*AD*CH*English*ECE	0.206897	0.172414	1	3, 6, 9, 20, 55, 60
**6**	D*me*AD*CH*LOTE*ece	0.0689655	0.0344828	1	47 , 79
**7**	D *ME*AD *r*LOTE*ece	0.0689655	0.0344828	1	54 , 61
**8**	D *ME *RS*CH*LOTE* ECE	0.0689655	0.0689655	1	30 , 69
**9**	D*me*RS*CH *English* ECE	0.103448	0.0689655	1	9 , 25 , 72
**10**	d*me *ad*rs*ch*English*ece	0.0344828	0.0344828	1	35

**11**	d* me*ad*rs*CH*LOTE* ECE	0.0344828	0.0344828	1	1
**12**	D *ME*ad *rs*ch *LOTE*ece	0.0344828	0.0344828	1	24
**13**	D*ME*ad*rs*ch*LOTE* ECE	0.0344828	0.0344828	1	41
**solution coverage: 1; solution consistency:1.**					

4.1.3 GL Tests of Sufficiency: Parsimonious Solution

****

TRUTH TABLE ANALYSIS

****

File: F:/PhD/Data/MECSH Papers/Trial 7 QCA comparison only no conflicts.csv
Model: GoodLanguageOutcome = f(PreschoolDevelopment, MaternalEducation, Maternaldistressantenatally, Responsivity, Childreninhome, LOTE, Early Childhood Education)
Algorithm: Quine-McCluskey

--- PARSIMONIOUS SOLUTION ---
Frequency cut off: 1; Consistency cut off: 1
Cases with greater than 0.5 membership in term:

Path	Formula	Coverage		Consistency	Cases
		**Raw**	**Unique**		
**1**	RS	0.586207	0.0689655	1	9, 13, 18, 21, 24, 25, 28, 30, 46, 55, 60, 66, 68, 69, 71, 72, 79
**2**	d*me	0.172414	0.0344828	1	1 , 13 , 35 , 47 , 79
**3**	me*CH	0.37931	0.0689655	1	1 , 3 , 6 , 9 , 13 , 25 , 46 , 47 , 71 , 72 , 79
**4**	D*ME	0.517241	0.241379	1	20, 21, 24, 26, 28, 30, 41, 51, 54, 55, 57, 60, 61, 68, 69
**solution coverage: 1; solution consistency:1.**					

4.2 Poor Language Outcome

4.2.1 PL Necessary Conditions

****

TRUTH TABLE ANALYSIS

****

Conditions tested:	Consistency	Coverage
**Preschool Development**	0.400000	0.076923
**~Preschool Development**	0.600000	0.375000
**Maternal Education**	0.400000	0.166667
**~Maternal Education**	0.600000	0.125000
**Maternal distress antenatally**	0.400000	0.090909
**~Maternal distress antenatally**	0.600000	0.250000
**Responsivity**	0.000000	0.000000
**~Responsivity**	1.000000	0.294118
**Children in home**	0.200000	0.043478
**~Children in home**	0.800000	0.363636

**English**	0.200000	0.052632
**~LOTE**	0.800000	0.266667
**Early Childhood Education**	0.400000	0.133333
**~Early Childhood Education**	0.600000	0.157895

4.2.2 PL Tests of Sufficiency: Complex Solution

File: F:/PhD/Data/MECSH Papers/Trial 7 QCA compariosn only no conflicts.csv
Model: ~GoodLanguageOutcome = f(PreschoolDevelopment, MaternalEducation, Maternaldistressantenatally, Responsivity, Childreninhome, LOTE, Early Childhood Education)
Algorithm: Quine-McCluskey

Frequency cut off: 1; Consistency cut off: 1
Cases with greater than 0.5 membership in term:

Path	Formula	Coverage		Consistency	Cases
		**Raw**	**Unique**		
**1**	d*ME*ad *r*ch*LOTE	0. 4	0.4	1	45, 52
**2**	D*me*ad*rs*ch*LOTE*ece	0.2	0.2	1	31
**3**	d*ME*AD*rs*CH*LOTE*ece	0.2	0.2	1	64
**4**	D*me*AD*rs*ch*English*ECE	0. 2	0.2	1	38
**solution coverage: 1; solution consistency: 1**					

4.2.3 PL Tests of Sufficiency: Parsimonious Solution

****
TRUTH TABLE ANALYSIS
****

File: F:/PhD/Data/MECSH Papers/Trial 7 QCA compariosn only no conflicts.csv
Model: ~GoodLanguageOutcome = f(PreschoolDevelopment, MaternalEducation, Maternaldistressantenatally, Responsivity, Childreninhome, LOTE, Early Childhood Education)
Algorithm: Quine-McCluskey

frequency cutoff: 1; consistency cutoff: 1

Path	Formula	Coverage		Consistency	Cases
		**Raw**	**Unique**		
**1**	d*ME	0. 6	0.6	1	45, 52, 64
**2**	D*me*rs*ch	0.4	0.4	1	31, 38
**solution coverage: 1; solution consistency: 1**					

References

Arcos Holzinger, L., & Biddle, N. (2015). *The relationship between early Childhood Education and Care (ECEC) and the outcomes of Indigenous childre: Evidence from the Longitudinal sttudy of Indigenous Children (LSIC)*. Retrieved from Canberra: Australia: http://caepr.cass.anu.edu.au/research/publications/relationship-between-early-childhood-education-and-care-ecec-and-outcomes

Baydar, N., Kuntay, A. C., Yagmurlu, B., Aydemir, N., Cankaya, D., Goksen, F., & Cemalcilar, Z. (2014). "It takes a village" to support the vocabulary development of children with multiple risk factors. *Developmental Psychology, 50*(4), 1014-1025.

Bayley, N. (1993). *Bayley Scales of Infant Development* (2nd edition. ed.). San Antonio, TX: Psychological Corp.

Belsky, J., Vandell, D. L., Burchinal, M., Clarke-Stewart, K. A., McCartney, K., Owen, M. T., & Network, N. E. C. C. R. (2007). Are there long-term effects of early child care? *Child Development, 78*(2), 681-701.

Bornstein, M. H., Tamis-LeMonda, C. S., Hahn, C.-S., & Haynes, O. (2008). Maternal responsiveness to young children at three ages: Longitudinal analysis of a multidimensional, modular, and specific parenting construct. *Developmental Psychology, 44*(3), 867-874.

Caldwell, B. M., & Bradley, R. H. (2003). *Home inventory administration manual*: University of Arkansas for Medical Sciences.

Christensen, D., Taylor, C. L., & Zubrick, S. R. (2017). Patterns of multiple risk exposures for low receptive vocabulary growth 4-8 years in the longitudinal study of Australian children. *PLoS ONE, 12*(1), e0168804.

Christensen, D., Zubrick, S. R., Lawrence, D., Mitrou, F., & Taylor, C. L. (2014). Risk Factors for Low Receptive Vocabulary Abilities in the Preschool and Early School Years in the Longitudinal Study of Australian Children. *PLoS ONE, 9*(7), e101476.

Durand, V. N., Loe, I. M., Yeatman, J. D., & Feldman, H. M. (2013). Effects of early language, speech, and cognition on later reading: a mediation analysis. *Frontiers in Psychology, 4*, 586. doi:10.3389/fpsyg.2013.00586

Evans, G. W., Boxhill, L., & Pinkava, M. (2008). Poverty and maternal responsiveness: The role of maternal stress and social resources. *International Journal of Behavioral Development, 32*(3), 232-237.

Grace, S. L., Evindar, A., & Stewart, D. (2003). The effect of postpartum depression on child cognitive development and behavior: a review and critical analysis of the literature. *Archives of Women's Mental Health, 6*(4), 263-274.

Harding, J. F., Morris, P. A., & Hughes, D. (2015). The relationship between maternal education and children's academic outcomes: A theoretical framework. *Journal of Marriage and Family, 77*(1), 60-76.

Harrison, L., & McLeod, S. (2010). Risk and protective factors associated with speech and language impairment in a nationally representative sample of 4- to 5-year-old children. *Journal of Speech, Language, and Hearing Research, 53*(2), 508-529.

Hirsh-Pasek, K., Adamson, L. B., Bakeman, R., Owen, M. T., Golinkoff, R. M., Pace, A., . . . Suma, K. (2015). The contribution of early communication quality to low-income children's language success. *Psychological Science, 26*(7), 1071-1083.

Hoff, E. (2006). How social contexts support and shape language development. *Developmental review, 26*(1), 55-88.

Hoff, E. (2018). Bilingual development in children of immigrant families. *Child development perspectives, 12*(2), 80-86.

Johnson, S. B., Riis, J. L., & Noble, K. G. (2016). State of the art review: Poverty and the developing brain. *Pediatrics, 137*(4), 1-16.

Kemp, L., Harris, E., McMahon, C., Matthey, S., Vimpani, G., Anderson, T., . . . Zapart, S. (2011). Child and family outcomes of a long-term nurse home visitation programme: a randomised controlled trial. *Archives of disease in childhood*, archdischild196279.

Kim, S., Im, H., & Kwon, K.-A. (2015). *The role of home literacy environment in toddlerhood in development of vocabulary and decoding skills.* Paper presented at the Child & Youth Care Forum.
Law, J., Plunkett, C. C., & Stringer, H. (2012). Communication interventions and their impact on behaviour in the young child: A systematic review. *Child Language Teaching and Therapy, 28*(1), 7-23. doi:http://dx.doi.org/10.1177/0265659011414214
Law, J., Rush, R., Parsons, S., & Schoon, I. (2013). The relationship between gender, receptive vocabulary, and literacy from school entry through to adulthood. *International Journal of Speech-Language Pathology, 15*(4), 407-415.
Levickis, P., Reilly, S., Girolametto, L., Ukoumunne, O. C., & Wake, M. (2014). Maternal behaviors promoting language acquisition in slow-to-talk toddlers: Prospective community-based study. *Journal of Developmental and Behavioral Pediatrics, 35*(4), 274-281.
McKean, C., Mensah, F. K., Eadie, P., Bavin, E. L., Bretherton, L., Cini, E., & Reilly, S. (2015). Levers for Language Growth: Characteristics and Predictors of Language Trajectories between 4 and 7 Years. *PLoS ONE, 10*(8), e0134251.
McKean, C., Wraith, D., Eadie, P., Cook, F., Mensah, F., & Reilly, S. (2017). Subgroups in language trajectories from 4 to 11 years: the nature and predictors of stable, improving and decreasing language trajectory groups. *Journal of Child Psychology and Psychiatry, 58*(10), 1081-1091. doi:10.1111/jcpp.12790
Melvin, S. A., Brito, N. H., Mack, L. J., Engelhardt, L. E., Fifer, W. P., Elliott, A. J., & Noble, K. G. (2017). Home environment, but not socioeconomic status, is linked to differences in early phonetic perception ability. *Infancy, 22*(1), 42-55. doi:http://dx.doi.org/10.1111/infa.12145
Milgrom, J., Westley, D. T., & Gemmill, A. W. (2004). The mediating role of maternal responsiveness in some longer term effects of postnatal depression on infant development. *Infant Behavior & Development, 27*(4), 443-454.
Murray, L., Arteche, A., Fearon, P., Halligan, S., Croudace, T., & Cooper, P. (2010). The effects of maternal postnatal depression and child sex on academic performance at age 16 years: a developmental approach. *Journal of Child Psychology and Psychiatry, 51*(10), 1150-1159.
Noble, K. G., Engelhardt, L. E., Brito, N. H., Mack, L. J., Nail, E. J., Angal, J., . . . Elliott, A. J. (2015). Socioeconomic disparities in neurocognitive development in the first two years of life. *Developmental Psychobiology, 57*(5), 535-551. doi:http://dx.doi.org/10.1002/dev.21303
Paavola, L., Kunnari, S., & Moilanen, I. (2005). Maternal responsiveness and infant intentional communication: Implications for the early communicative and linguistic development. *Child: Care, Health and Development, 31*(6), 727-735.
Pearson, R., Melotti, R., Heron, J., Joinson, C., Stein, A., Ramchandani, P., & Evans, J. (2012). Disruption to the development of maternal responsiveness? The impact of prenatal depression on mother-infant interactions. *Infant Behavior & Development, 35*(4), 613-626.
Piccolo, L. R., Merz, E. C., He, X., Sowell, E. R., & Noble, K. G. (2016). Age-related differences in cortical thickness vary by socioeconomic status. *PLoS ONE Vol 11(9), 2016, ArtID e0162511, 11*(9).
Ribot, K. M., Hoff, E., & Burridge, A. (2018). Language use contributes to expressive language growth: Evidence from bilingual children. *Child Development, 89*(3), 929-940.
Sénéchal, M., Whissell, J., Bildfell, A., Cain, K., Compton, D., & Parrila, R. (2017). Starting from home: Home literacy practices that make a difference. *Theories of reading development*, 383-408.
Shonkoff, J. P., Garner, A. S., Child, C. o. P. A. o., Health, F., Committee on Early Childhood, A., & Care, D. (2011). The lifelong effects of early childhood adversity and toxic stress. *Pediatrics*, peds. 2011-2663.
Short, K., Eadie, P., Descallar, J., Comino, E., & Kemp, L. (2017). Longitudinal vocabulary development in Australian urban Aboriginal children: Protective and risk factors. *Child: Care, Health and Development, 43*(6), 906-917.
Smith, K. E., Landry, S. H., & Swank, P. R. (2006). The role of early maternal responsiveness in supporting school-aged cognitive development for children who vary in birth status. *Pediatrics, 117*(5), 1608-1617.
Sylva, K., Melhuish, E., Sammons, P., Siraj-Blatchford, I., Taggart, B., Smees, R., . . . Morahan, M. (2004). The effective provision of pre-school education (EPPE) project.

Taggart, B., Sylva, K., Melhuish, E., Sammons, P., & Siraj, I. (2015). Effective pre-school, primary and secondary education project (EPPSE 3-16+): How pre-school influences children and young people's attainment and developmental outcomes over time.

Talge, N. M., Neal, C., Glover, V., Early Stress, T. R., Fetal, P. S. N., Child, N. E. o., & Health, A. M. (2007). Antenatal maternal stress and long-term effects on child neurodevelopment: how and why? *Journal of Child Psychology and Psychiatry, 48*(3-4), 245-261.

Tamis-LeMonda, C. S., Kuchirko, Y., & Song, L. (2014). Why is infant language learning facilitated by parental responsiveness? *Current Directions in Psychological Science, 23*(2), 121-126.

Tayler, C. (2016). *The E4Kids study: Assessing the effectiveness of Australian early childhood education and care programs: overview of findings at 2016*. Retrieved from Melbourne, Australia: https://education.unimelb.edu.au/news_and_activities/projects/E4Kids

Taylor, C. L., Christensen, D., Lawrence, D., Mitrou, F., & Zubrick, S. R. (2013). Risk Factors for Children's Receptive Vocabulary Development from Four to Eight Years in the Longitudinal Study of Australian Children. *PLoS ONE, 8*(9), e73046.

Taylor, H. B., Anthony, J. L., Aghara, R., Smith, K. E., & Landry, S. H. (2008). The interaction of early maternal responsiveness and children's cognitive abilities on later decoding and reading comprehension skills. *Early Education and Development, 19*(1), 188-207.

Tomblin, J. B., Hardy, J. C., & Hein, H. A. (1991). Predicting poor-communication status in preschool children using risk factors present at birth. *Journal of Speech, Language, and Hearing Research, 34*(5), 1096-1105.

Tough, S. C., Siever, J. E., Leew, S., Johnston, D. W., Benzies, K., & Clark, D. (2008). Maternal mental health predicts risk of developmental problems at 3 years of age: follow up of a community based trial. *BMC pregnancy and childbirth, 8*(1), 16.

Ursache, A., & Noble, K. G. (2016). Neurocognitive development in socioeconomic context: Multiple mechanisms and implications for measuring socioeconomic status. *Psychophysiology, 53*(1), 71-82. doi:http://dx.doi.org/10.1111/psyp.12547

Van den Bergh, B. R., Mulder, E. J., Mennes, M., & Glover, V. (2005). Antenatal maternal anxiety and stress and the neurobehavioural development of the fetus and child: links and possible mechanisms. A review. *Neuroscience & Biobehavioral Reviews, 29*(2), 237-258.

Wechsler, D. (2002). *Wechsler Preschool and Primary Scale of Intelligence (WPPSI-III)* (Third edition ed.). San Antonio, TX: Harcourt Asessment. Inc.

Zubrick, S. R., Taylor, C. L., Rice, M. L., & Slegers, D. W. (2007). Late language emergence at 24 months: An epidemiological study of prevalence, predictors, and covariates. *Journal of Speech, Language, and Hearing Research, 50*(6), 1562-1592.
